# The eukaryotic homology search complex distorts donor DNA structure to probe for homology

**DOI:** 10.1101/gad.353376.125

**Published:** 2026-06-01

**Authors:** Mitchell V. Woodhouse, Jingyi Hu, Meiling Wu, Jin Qian, James T. Inman, Michelle D. Wang, J. Brooks Crickard

**Affiliations:** 1 Deparment of Molecular Biology and Genetics, Cornell University, Ithaca, New York 14853, USA;; 2 Howard Hughes Medical Institute, Cornell University, Ithaca, New York 14853, USA;; 3 Laboratory of Atomic and Solid State Physics, Department of Physics, Cornell University, Ithaca, New York 14853, USA

**Keywords:** homologous recombination, DNA strand exchange, Rad54, Rad51, single molecule, optical trap, magnetic tweezers

## Abstract

Homologous recombination (HR) is a DNA double-strand break repair pathway that facilitates genetic exchange and protects damaged replication forks during DNA synthesis. As a template-based repair process, the successful repair of a double-strand break depends on locating suitable homology from a donor DNA sequence elsewhere in the genome. In eukaryotes, Rad51 catalyzes the homology search in coordination with the ATP-dependent motor protein Rad54. The mechanism by which these two proteins regulate forces on dsDNA substrates during homology search remains unknown. Here, we have utilized single-molecule magnetic tweezers and optical trapping methods to monitor remodeling of the DNA template during the homology search. We found that the activity of Rad51 and Rad54 remodels the donor DNA substrate to control the association and dissociation of Rad51–ssDNA filaments in the absence of DNA homology. The mechanism is through the generation of short, isolated, underwound DNA loops extruded by the homology search complex composed of Rad51 and Rad54. Finally, failure of Rad54 to act processively disrupts target selection in vivo. This study provides a basic understanding of how motorized homology search manipulates the donor DNA during the search for a suitable repair template.

## Abstract (precis)

In this study, Woodhouse et al. combine molecular tweezers and optical trapping to describe the mechanics of loop extrusion and extension that allow for homology searching during homologous recombination-mediated DNA repair. Rad51 and Rad54—the presynaptic complex—cooperatively and processively remodel the DNA template to form short DNA loops that facilitate the identification of DNA sequence homology.

Homologous recombination (HR) is a universally conserved template-based DNA double-strand break repair (DSBR) pathway that requires a recipient ssDNA to locate a matching donor DNA elsewhere in the genome (San Filippo et al. 2008; Jasin and Rothstein 2013; Kowalczykowski 2015). Finding a suitable donor involves a homology search. This systematic effort initially searches sequences local to the break site and then can extend to more distal regions until homology is located (Haber 2018; Piazza et al. 2021; Dumont et al. 2024). Members of the RecA recombinase family control the selection and stabilization of the donor DNA sequence (Forget and Kowalczykowski 2012; Ragunathan et al. 2012; Renkawitz et al. 2013; Lee et al. 2015; Qi et al. 2015; Bell and Kowalczykowski 2016; Greene 2016; Wiktor et al. 2021; Yang and Pavletich 2021; Danilowicz et al. 2024).

The RecA family of recombinases is conserved in all domains of life. The basic biochemical mechanism of these proteins involves the formation of a filament on the single-stranded recipient DNA, where it binds and stabilizes the ssDNA at 1.5 times the contour length of B-form DNA (Yang et al. 2020; Yang and Pavletich 2021). The 3 nt spacing of individual protomers in this filament enables the coordination of two DNA-binding sites that can stretch the donor duplex, promoting the kinetic sampling of base pairs (Qi et al. 2015). Binding site I is composed of DNA-binding loops from distinct RecA protomers that allow the unstacking of bases from the parent donor duplex (Xu et al. 2017; Yang et al. 2020; Yang and Pavletich 2021) and promote base flipping to enhance contacts between the incoming recipient and the donor DNA. A second DNA-binding site, DNA-binding site II, interacts with the nonhomologous strand, stabilizing the separation of the two parent strands (Cloud et al. 2012). The two binding sites coordinate during the homology search to actively probe the donor DNA. A minimum of eight paired nucleotides is required for stable kinetic sampling (Qi et al. 2015; Bell and Kowalczykowski 2016; Greene 2016). A spacing of four recombinase protomers, resulting in 12 paired nucleotides, is optimal for stable sequence selection (De Vlaminck et al. 2012; Xu et al. 2017). Further recipient donor pairing is considered a strand exchange reaction, leading to a displacement loop (D-loop).

Both DNA binding sites I and II favor ssDNA binding (De Vlaminck et al. 2012), making dsDNA a poor substrate for Rad51–ssDNA filaments. Partial separation of DNA strands can promote the binding of recombinase filaments in the absence of DNA sequence homology, as the duplex DNA begins to resemble single-stranded DNA (ssDNA). Underwinding of DNA can lead to partial strand separation by reducing the number of bases per turn of the B-form helix. Mechanically, this can be achieved by rotating the DNA around the superhelical axis, which adds torsion (Strick et al. 2000; Sheinin et al. 2011; Xu et al. 2021). It has been experimentally determined that the addition of forces by stretching (Vlassakis et al. 2013; Danilowicz et al. 2014) or rotating (van der Heijden et al. 2008; De Vlaminck et al. 2012) DNA can improve the binding of recombinase–ssDNA filaments to the donor DNA in the absence of DNA sequence homology. The superhelical density of DNA in vivo is generally underwound, improving recombinase filament binding. However, this is not uniform, which makes regulation of DNA topology a crucial feature in controlling the binding of recombinase filaments to the donor DNA during the homology search. The factors that aid recombinases in the homology search may influence early DNA sequence recognition by providing forces that regulate topology, but the mechanism underlying this is unclear.

In eukaryotes, the ATP-dependent translocase Rad54 is thought to aid Rad51 during the homology search and strand exchange (Solinger et al. 2001; Wolner et al. 2003; Renkawitz et al. 2013, 2014; Tavares et al. 2019; Crickard et al. 2020b; Sridalla et al. 2024). However, this relationship remains somewhat controversial (Sugawara et al. 2003; Wolner and Peterson 2005). Evidence suggests that Rad51 can identify short stretches of sequence homology and form unstable joints in the absence of Rad54 (Sinha and Peterson 2008). Rad54 then converts these unstable products into a stable strand exchange intermediate, a displacement loop (D-loop). This model remains consistent with Rad54's role in the homology search, as sequence site selection requires identifying the substrate and establishing stable binding. These two steps can be viewed as distinct, requiring different amounts of energy (Mirny et al. 2009), and both can be considered mechanical steps in the homology search. Based on our current understanding, this would allow Rad51 to facilitate the first step and Rad54 to drive the second. The mechanism behind conversion to a stable joint molecule remains incompletely understood. Current models suggest that Rad54 may remove Rad51 from newly formed joints, which allows relaxation of helical tension on the newly paired recipient–donor substrate (van Mameren et al. 2009; Wright and Heyer 2014). However, an alternate model suggests that the addition of negative supercoils to the donor DNA substrate may help promote conversion to a stable product (Van Komen et al. 2000; Ristic et al. 2001; Crickard et al. 2020b).

Rad51 enhances the ATP hydrolysis and translocation activity of Rad54, and together they can form a complex (Petukhova et al. 1998, 1999; Mazin et al. 2000; Solinger et al. 2001; Crickard et al. 2020b). The interaction between Rad54 and Rad51 occurs through Rad54's intrinsically disordered N-terminal domain (Alexiadis et al. 2004; Raschle et al. 2004). This region can interact with Rad51 and form a multimerization domain with other Rad54 molecules. Removal or replacement of the N-terminal domain renders *Saccharomyces cerevisiae* cells sensitive to DNA-damaging agents, comparable with the *rad54*Δ strains (Alexiadis et al. 2004; Raschle et al. 2004; Wright and Heyer 2014; Crickard et al. 2020a). Rad54 is related to chromatin-remodeling enzymes of the Snf2 family (Flaus et al. 2006) and has also been shown to have nucleosome-remodeling activity (Alexeev et al. 2003; Wolner and Peterson 2005; Zhang et al. 2007; Crickard et al. 2020b). The fundamental activity of Rad54 is to physically move along dsDNA, adding twist to the DNA superhelical axis (Jaskelioff et al. 2003; Thomä et al. 2005; Amitani et al. 2006; Hopfner et al. 2012). The magnitude of these remodeling events has not been measured. However, this activity can result in the removal of other proteins from DNA or the storage of a negative twist to the DNA's superhelical axis. The accumulation of supercoils in DNA requires regions to be topologically locked. Recent evidence has emerged that negative supercoils may accumulate even on linear DNA. The mechanism behind this is unknown.

Here, we used single-molecule methods to investigate how the Rad51–Rad54 complex, known as the presynaptic complex (PSC), can form underwound DNA loops. These loops form through Rad54-mediated translocation and occur before the recognition of DNA sequence homology. The resulting underwound DNA stabilizes Rad51–ssDNA binding, and we demonstrated that failure to form loops results in D-loop formation failure in vivo. From this, we developed a hypothesis that the formation of underwound DNA loops before the identification of DNA sequence homology predisposes the PSC to convert primary sequence identification events into stably paired products by promoting Rad51–ssDNA stability on donor DNA.

## Results

### PSC activity is dependent on the tension placed on the donor DNA

By tracking the minor groove of dsDNA, Rad54 introduces helical turns to the DNA backbone (Ceballos and Heyer 2011; Hopfner et al. 2012). The torsional stress generated by translocation accumulates only if it occurs in a topologically locked region of donor DNA (Van Komen et al. 2000; Ristic et al. 2001; Tan et al. 2003; Sanchez et al. 2013; Crickard et al. 2020b). A locked region forms when a group of proteins establishes multiple contacts with the DNA. In this scenario, one contact can pump DNA into an isolated loop, while another serves as an anchor. A consequence of this mechanism would be the apparent ability to extrude loops, resulting in DNA compaction. Previous reports have shown that the Rad54 paralog Rdh54 can move along dsDNA by loop extrusion or directional translocation (Prasad et al. 2007). Rad54 or the combination of Rad54, Rad51, and ssDNA, known as the presynaptic complex (PSC) (Crickard et al. 2020b; Sridalla et al. 2024), has not been observed to promote loop extrusion.

**Figure 1.**
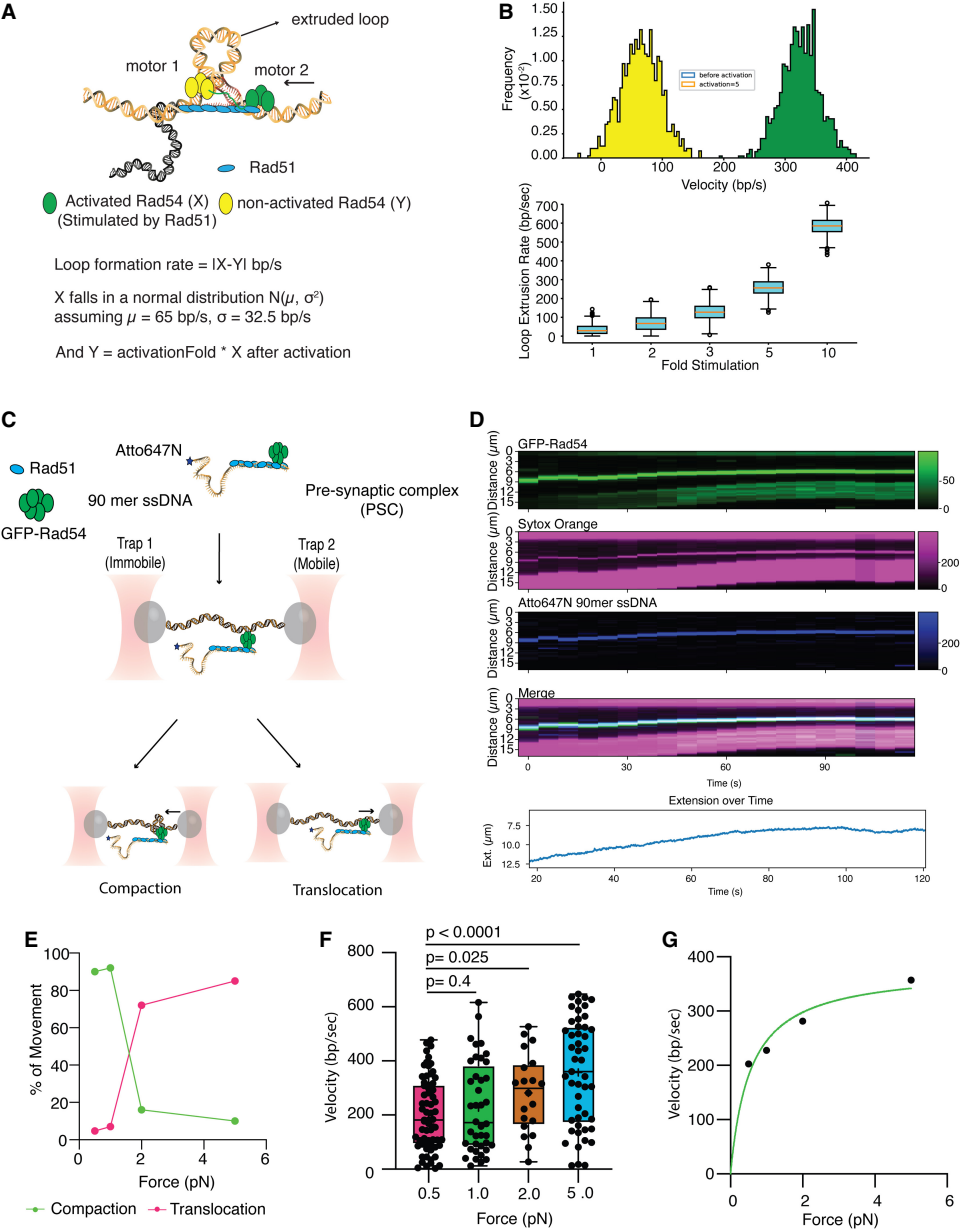
The PSC switches from compaction to translocation at higher force. (*A*) A general model for how Rad54 could work as a dimer to promote loop extrusion. The model uses a measured rate of 65 bp/sec of nonactivated Rad54 to predict loop extrusion rates, considering fold stimulation of a second motor. (*B*, *top*) Simulation of Rad54 velocities with and without stimulation. (*Bottom*) The normally distributed data were then used to estimate a loop extrusion rate that depends solely on the fold activation of Rad54. (*C*) Diagram illustrating the design to measure the real-time activity of the PSC. (*D*) Representative kymographs for PSC activity at 0.5 pN. (*Top*) GFP-Rad54. (Second from *top*) Sytox orange donor DNA. (Second from *bottom*) Atto647N 90-mer ssDNA. (*Bottom*) Merged. *Below* the kymographs is an extension curve taken during the experiment. (*E*). Graph representing the percentage of molecules that undergo compaction at 0.5 pN (*N* = 19/20), 1.0 pN (*N* = 13/14), 2.0 pN (*N* = 3/16), and 5.0 pN (*N* = 2/17) or translocation at 0.5 pN (*N* = 1/20), 1.0 pN (*N* = 1/14), 2.0 pN (*N* = 13/16), and 5.0 pN (*N* = 17/19). (*F*) Box plots illustrate the rate of PSC activity at 0.5 pN (*N* = 66) and 1.0 pN (*N* = 40) (*left*) and at 2.0 pN (*N* = 20) and 5.0 pN (*N* = 49) (*right*). The “+” shows the mean, and the error bars indicate the data range. (*G*) Fit of the velocity data at 0.5, 1.0, 2.0, and 5.0 pN; the data were fit by a rectangular hyperbola (*r*^2^ = 0.9). The significance of all differences was calculated using an unpaired *t*-test.

We developed a simple model for how Rad54 may stimulate loop extrusion, because Rad51 stimulates the Rad54 activity by threefold to fivefold (Mazin et al. 2000). We assumed that the rate at which any loop would grow is proportional to the combined rates of the two Rad54 subunits contacting the DNA (Fig. 1A). If one of these subunits was activated by Rad51 and the other was not, then the loop should grow at a defined rate dependent on the fold stimulation of the activated subunit. We used the previously measured Rad54 translocation rate (65 bp/sec) as the slow (anchoring motor) rate and simulated activated translocation rates at onefold, twofold, threefold, fivefold, and 10-fold stimulation (Fig. 1B). This generated mean loop extrusion rates of 150 and 250 bp/sec at threefold and fivefold stimulation, respectively (Fig. 1B), rates that have been documented for Rad51–Rad54 activity.

To test this model, we used a dual optical trap with confocal microscopy to monitor the binding of the PSC to dsDNA. We formed the PSC with GFP-Rad54 in combination with Atto647N 90-mer ssDNA bound by Rad51, a strategy that we have employed previously to monitor active PSCs (Crickard et al. 2020a,b; Sridalla et al. 2024). Application of a constant force via a force clamp controlled the flexibility of the donor dsDNA, allowing us to test the hypothesis that the PSC can compact DNA and measure the specific compaction rate. We measured the PSC's activity at forces of 0.5, 1.0, 2.0, and 5 pN (Fig. 1C–E). The PSC compacted DNA at 0.5 and 1.0 pN (Fig. 1E) and transitioned to movement along the DNA without compacting at 2.0 and 5.0 pN (Fig. 1E). The rates of compaction and movement were comparable, with the rate of compaction at 0.5 pN being 202 ± 132 bp/sec. The measured movement rate at 2 pN was 272.6 ± 133 bp/sec, and the rate at 5 pN was 357 ± 195 bp/sec (Fig. 1F), suggesting that the motor was not inactivated at higher forces. We also observed an increase in the rate of linear movement at higher forces (Fig. 1F,G), suggesting that the motor was converting more energy into linear translocation. Interestingly, the translocation rate at 5 pN approached estimates from our model for a fivefold stimulated Rad54 activity in the absence of a trailing motor. Similar observations have been made for other chromatin-remodeling enzymes—ySwi/Snf and yRSC (Havas et al. 2000; Zhang et al. 2006)—as linear movement becomes decoupled from the stabilization of added helical turns to the DNA. However, in the case of the PSC, loop formation occurs significantly faster, and the loops are more stable. This is likely due to the PSC's unique structural configuration and is consistent with our model.

**Figure 2.**
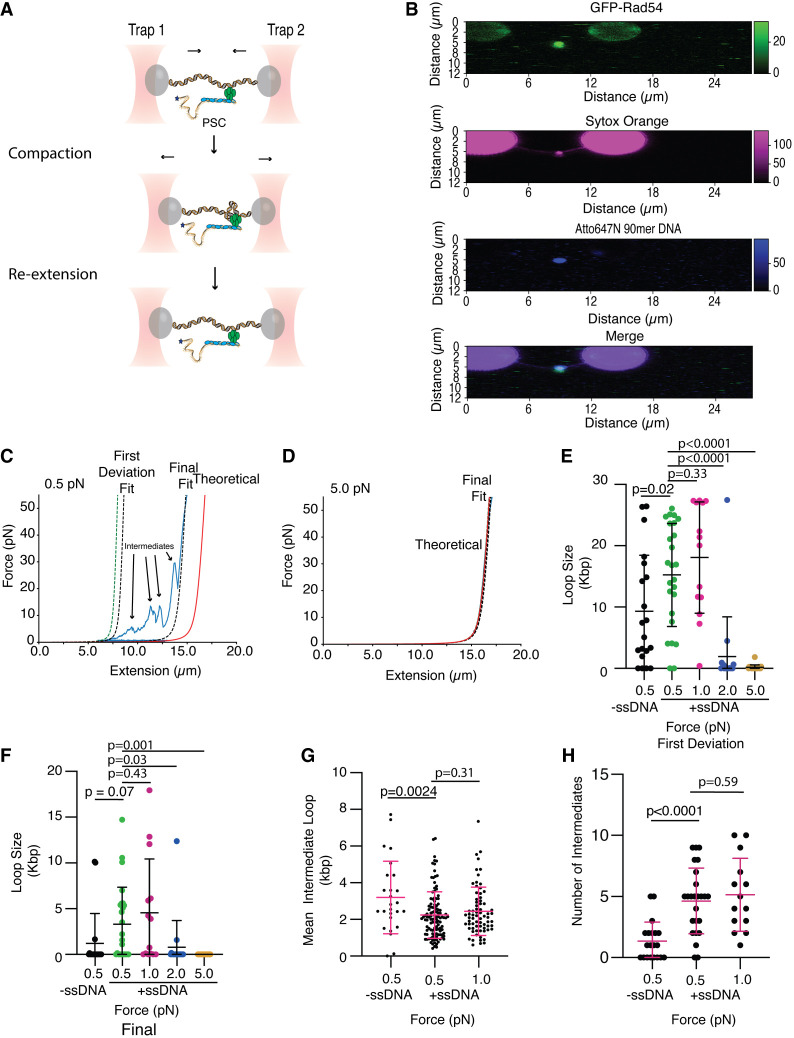
The PSC compacts donor DNA in a force-dependent manner. (*A*) Diagram of the optical trapping experiment designed to determine whether compaction generates multiple points of contact. (*B*) Representative widefield images of GFP-Rad54 (*top*), Sytox orange donor DNA (second from *top*), and Atto647N 90-mer ssDNA (second from *bottom*) and a merged image (*bottom*). (*C*) Force extension (FE) curve for the re-extension of a DNA molecule compacted by the PSC at 0.5 pN. The theoretical line is in red, and the data are in blue. The dashed lines represent fits to the traces. The green dashed line is the initial point of deviation of the theoretical FE curve. (*D*) Force extension curve for the re-extension of a DNA molecule compacted by the PSC at 5.0 pN. The theoretical line is in red, and the data are in blue. The dashed lines represent fits to the traces. (*E*) Graph illustrating the degree of DNA compaction at the first deviation from theoretical for 0.5 pN −ssDNA (*N* = 20) and +ssDNA (*N* = 25) and for 1.0 pN (*N* = 14), 2.0 pN (*N* = 18), and 5.0 pN (*N* = 20). The bar represents the mean, and the error bars indicate the standard deviation of the data. (*F*) Graph illustrating the degree of DNA compaction at the final deviation from theoretical for 0.5 pN −ssDNA (*N* = 18) and +ssDNA (*N* = 25) and for 1.0 pN (*N* = 14), 2.0 pN (*N* = 18), and 5.0 pN (*N* = 18). The bars represent the mean, and the error bars indicate the standard deviation of the data. (*G*) Graph representing the mean intermediate loop size at 0.5 pN −ssDNA (*N* = 26) and +ssDNA (*N* = 115) and at 1.0 pN (*N* = 71). The bars represent the mean, and the error bars indicate the standard deviation of the data. (*H*) Graph representing the number of intermediates per tether at 0.5 pN −ssDNA (*N* = 20) and +ssDNA (*N* = 25) and at 1.0 pN (*N* = 14). The bar represents the mean, and the error bars indicate the standard deviation of the experiment. All statistical significance was determined using an unpaired *t*-test.

A necessary condition for the model is that the PSC can make multiple contacts with the donor DNA. This could occur as a single large loop or a series of smaller loops. We tested these two scenarios by allowing the PSC to compact DNA and then re-extend the DNA by separating the optical trap at a constant rate (Fig. 2A,B). If compaction was due to multiple points of contact, then re-extension of the compacted DNA should result in a sawtooth pattern as contact points are disrupted (Baumann et al. 2000; Le et al. 2023). Disruption of contacts could occur through direct loss of the physical interaction between the protein and DNA or through the sliding of the protein along the DNA, resulting in a slip–stick mechanism. For simplicity, we viewed both mechanisms as potential disruption events. When we pulled on the compacted DNA molecules at 0.5 and 1.0 pN, they displayed sawtooth patterns (Fig. 2C). As expected, this behavior was not observed at higher forces because there was no compaction, and all molecules followed a theoretical force extension (FE) curve consistent with B-form DNA (Fig. 2D).

The initial amount of compacted DNA is defined as the first deviation from the theoretical FE curve and represents the initial amount of compacted DNA (Fig. 2C). We continued pulling on the DNA past this point and observed multiple intermediate deviations from the theoretical extension curve. These intermediates reflect numerous contact points between the PSC and the DNA (Fig. 2C). Structures formed at 0.5 and 1.0 pN of force initially constrained ∼15 kb of DNA. The final size of constrained DNA was ∼2 kb (Fig. 2E,F). The mean of the DNA isolated in intermediates was ∼2–2.5 kb per intermediate at both 0.5 and 1.0 pN (Fig. 2G). We observed a mean contact number of four to five intermediates per molecule (Fig. 2H). It should be noted that due to the small size of the isolated loops, we were unable to visualize them directly. However, based on these data, we conclude that the PSC can isolate individual donor DNA loops of ∼2 kb in length.

To determine whether these activities depended on ATP hydrolysis, we measured donor DNA compaction in the presence of AMPPNP at 0.5 pN. We did not observe processive compaction of the DNA. A few short loops did form, but these molecules had significantly smaller loops and fewer intermediate binding events (Supplemental Fig. S1A–C). From this, we conclude that compaction and linear translocation are dependent on ATP hydrolysis. We also performed experiments in which ssDNA was omitted from the PSC reconstitution. We reasoned that the multiple points of contact observed within the PSC could result from Rad51 filamentation. Therefore, we measured Rad51 and Rad54 activity in the absence of ssDNA. Surprisingly, the compaction velocity did not significantly decrease (Supplemental Fig. S1D). Likewise, there was no difference in the mean loop size that formed (Fig. 2G). However, we did observe significant differences in the size of the initial DNA isolated and the number of measured contact points per molecule (Fig. 2E,H, *P* = 0.02 and *P* < 0.0001, respectively). This was not due to an increase in the amount of GFP-Rad54 present, and all conditions showed similar GFP-Rad54 intensity (Supplemental Fig. S1E). Not surprisingly, this suggests that Rad51 and Rad54 are cooperative in the absence of ssDNA but that filament formation on ssDNA helps distribute this activity along the donor DNA.

### Rad54 applies a significant force during translocation

Our original model was based on a dimer of Rad54 driving loop extrusion and fit the loop formation rate. However, we observed multiple points of contact, suggesting more complex interactions that could be cooperative or antagonistic. Multimeric DNA motors could act in series or in parallel, depending on their structural organization. Therefore, to determine whether the multiple points of contact could work cooperatively, the PSC was loaded at extensions of 6, 8, 10, 12, and 14 µm of the donor DNA. These values represent 36%, 49%, 61%, 61%, 73%, and 81% of the full extension length of λ-DNA (16.4 µm). By loading the PSC at different extensions, a trend should be evident as the number of contacts between the PSC and donor DNA decreases.

**Figure 3.**
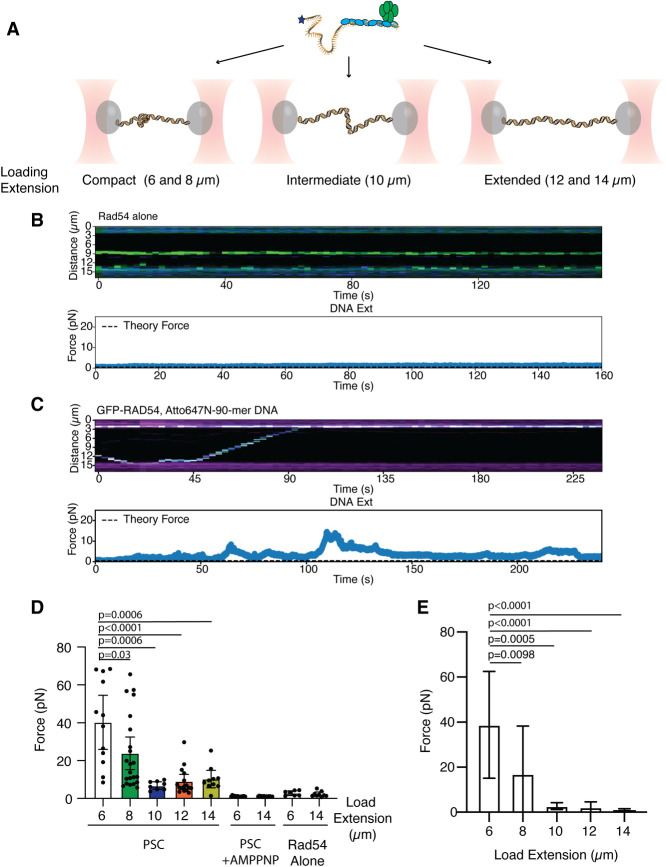
The PSC generates a significant force during translocation. (*A*) Illustration of the extension loading experiment. The PSC was loaded at different DNA extensions. (*B*) Representative kymograph and force trace for Rad54 alone. (*C*) Representative kymograph and force trace for the PSC. (*D*) Maximum force output during activity measurements for the PSC+ATP at 6 µm (*N* = 12), 8 µm (*N* = 22), 10 µm (*N* = 8), 12 µm (*N* = 16), and 14 µm (*N* = 10) loading extensions; for the PSC+AMPPMP at 6 µm (*N* = 12) and 14 µm (*N* = 13) loading extensions; and for Rad54 at 6 µm (*N* = 7) and 14 µm (*N* = 9) loading extensions. The bars represent the mean, and the error bars indicate the standard deviation of the data. (*E*) Graph representing the initial force output at 12.5 µm extension after the PSC was bound at different extensions. The bar represents the mean, and the error bars indicate the standard deviation of the data. All statistical significance was determined using an unpaired *t*-test.

To measure the force during translocation, the beads were moved to a 12.5 µm extension after loading (Fig. 3A). The extension was maintained to prevent compaction of the beads, allowing measurement of the force output as the PSC moves along the donor DNA (Fig. 3B,C). The output was not constant during translocation, and we measured the maximum force outputs during individual translocation events (Fig. 3B,C). PSCs loaded at 6 and 8 µm had mean maximum force outputs of 40 and 23 pN (Fig. 3D). This force was significantly greater (*P* < 0.0001) than the mean maximum forces measured after loading the complex at 10, 12, and 14 µm, which were ∼6–8 pN (Fig. 3D). These values were greater than those observed for Rad54 alone at 6 and 14 µm loads (Fig. 3D) and were dependent on ATP hydrolysis (Fig. 3D). From this, we conclude that the multiple points of contact with the PSC could act cooperatively during translocation.

We further investigated the higher forces generated at shorter loading extensions by measuring the initial force of PSC binding after loading at 6, 8, 10, 12, and 14 µm. The initial force is defined as the value measured when the beads were extended to 12.5 µm after loading. As expected, the most significant mean force, 38 ± 23 pN, was observed when the PSC was loaded at 6 µm (Fig. 3E). This dropped off at higher loading extensions (Fig. 3E). This change likely represents a decrease in the number of contact points between the PSC and DNA. The higher forces observed at shorter loading extensions may reflect the formation of higher-order structures, creating multiple points of contact that act in parallel and can search the donor DNA.

### Rad51 and Rad54 remodel isolated DNA

The PSC can isolate stretches of donor dsDNA. The impact on the DNA topology in this region is unclear. Previous work has used P1 nuclease cleavage (Van Komen et al. 2000) and RPA binding to measure partial DNA duplex melting during the homology search (Crickard et al. 2020b). To improve the resolution of these measurements and to understand how PSC activity affects the remodeling of donor DNA, we used a magnetic tweezer (MT) system (Le et al. 2019, 2023; Lee et al. 2023) to monitor Rad54 and PSC activity on torsionally constrained (TC) DNA. The experimental setup consisted of a single 12.7 kb piece of dsDNA attached to a magnetic bead at one end and to the surface of a flow chamber at the other. Both strands are connected to the bead and the surface, thereby torsionally constraining the DNA (Supplemental Fig. S2A; Le et al. 2023). The magnetic tweezer setup allows precise control and measurement of DNA remodeling.

For each experiment, the DNA is initially rotated through turns in either the positive or negative direction, generating a hat curve based on the bead's height (Supplemental Fig. S2A). The curve results from changes in bead height as turns are added to the DNA backbone. On either side of the hat curve, the DNA undergoes a buckling transition, lowering the height of the beads. This transition results from plectoneme formation and is a response to torsional stress (Supplemental Fig. S2A; Forth et al. 2008; Lee et al. 2023). The binding and activity of Rad54 can then alter the height of the bead. Events that can alter bead height include stretching the regular B-form helix or adding isolated turns to the DNA.

The chirality of DNA remodeling can be measured by observing changes in the direction of the bead height. For example, if activity is measured at +30 magnet turns, shortening of DNA extension indicates that Rad54 has underwound the DNA. In contrast, at −60 magnet turns, underwinding the DNA will add positive turns to the plectoneme, thereby increasing the extension (Supplemental Fig. S2A,B). Rad54 alone shortened the bead height at +30 turns and lengthened it at −60 turns, consistent with the addition of negative turns to the DNA (Supplemental Fig. S2B). These data qualitatively suggest that Rad54 promotes underwinding of the DNA. To measure protein activity, we analyzed the extension by averaging over a 5 sec sliding window. We measured the extension rate using the same method. Again, these values can be positive or negative. Here, we report only the positive values, as there was no significant difference between the two rates.

We measured changes in DNA extension, initially starting at −60 turns, for Rad54 concentrations of 25, 125, and 500 pM. The size of extension events increased with increasing Rad54 concentration. The increase was not observed when ATP was omitted from the reaction (Supplemental Fig. S2C). A mean change of 0.79 µm in extension was observed at the highest Rad54 concentration tested. Surprisingly, a slight change in extension occurred even in the absence of ATP, which likely stems from Rad54 binding (Supplemental Fig. S2C). Alternatively, these small changes could reflect measurement noise and serve as a natural baseline. A maximum mean rate of 0.307 µm/sec was observed at the highest concentration of Rad54 tested (Supplemental Fig. S2D). Again, there was a small change in the absence of ATP, but there was no increase in activity with increasing protein concentration (Supplemental Fig. S2D).

The lifetime of each bead extension event was measured by recording the time elapsed for events lasting >2.5 sec and maintaining an extension at least 3 standard deviations (SD) above the baseline. The individual data points were then fit to an exponential decay curve to determine the half-life in seconds (Supplemental Fig. S2E). There was no difference in the half-life of the events with increasing Rad54 concentrations or in the absence of ATP (Supplemental Fig. S2E). These data indicate that Rad54 can processively add negative turns to the DNA in the presence of ATP.

**Figure 4.**
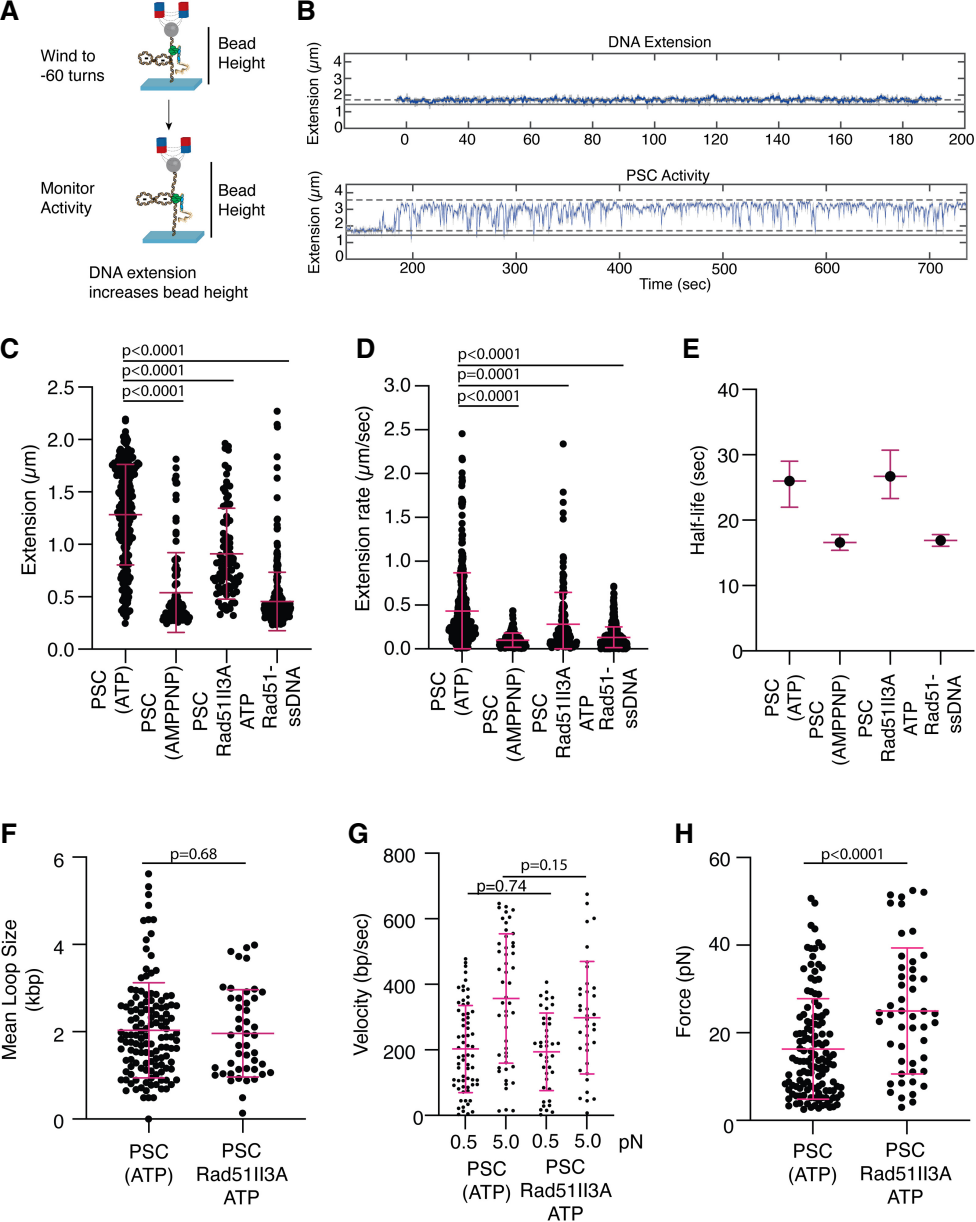
Full DNA extension requires ATP hydrolysis and Rad51–DNA binding site II. (*A*) Schematic of magnetic tweezer experiments with the PSC. (*B*) Activity trace for DNA and the PSC at −60 turns. The blue lines represent changes in bead height and in the extension of the DNA. (*Top*) The DNA extension was used as a baseline control to monitor deviation from DNA alone. The dashed lines represent the maximum and minimum extensions of the bead at −60 turns. (*C*) A graph representing the bead extension for 500 pM PSC (ATP; *N* = 265), 500 pM PSC (AMPPNP; *N* = 105), 500 pM PSC with Rad51-IIA (*N* = 82), and 5 nM Rad51–ssDNA (*N* = 309). The cross bar represents the mean of the data, and the error bars represent the standard deviation. (*D*) Graph representing the extensions per second for 500 pM PSC (ATP; *N* = 427), 500 pM PSC (AMPPNP; *N* = 144), 500 pM PSC with Rad51-IIA (*N* = 157), and 5 nM Rad51–ssDNA (*N* = 576). The bar represents the mean, and the error bars indicate the standard deviation. (*E*) Graph representing the half-life measurements for 500 pM PSC (ATP; *N* = 265), 500 pM PSC (AMPPNP; *N* = 105), 500 pM PSC with Rad51-IIA (*N* = 97), and 5 nM Rad51–ssDNA (*N* = 309). The dot represents the half-life, and the error bars indicate the 95% confidence interval of the fit. (*F*) Graph representing the mean loop size after compaction by the PSC (*N* = 130) and the PSC with Rad51-II3A (*N* = 46). The bar represents the mean, and the error bars indicate the standard deviation of the data. The PSC data are reproduced from Figure 2G. (*G*) Graph representing velocity for the PSC (*N* = 66 and *N* = 49) and the PSC with Rad51-II3A (*N* = 40 and *N* = 37) at 0.5 and 5.0 pN. The bar represents the mean, and the error bars indicate the standard deviation of the data. (*H*) A graph representing the force required to break contacts between the PSC and DNA for the PSC (*N* = 130) and the PSC with Rad51-IIA (*N* = 49). The bar represents the mean, and the error bars indicate the standard deviation of the data. As in Figure 2, the mean force is a measure of intermediate peaks in the re-extension curves. All statistical significance was tested using an unpaired *t*-test.

The activity of PSCs composed of Rad51, 90-mer ssDNA, and Rad54 was measured at an initial value of −60 turns (Fig. 4A,B) at two different concentrations (Supplemental Fig. S3A). Concentration-dependent differences were calculated by keeping the Rad54:Rad51:90-mer ssDNA ratio constant while varying the total concentration; for example, 500 pM Rad54 with 5 nM Rad51 and 125 pM Rad54 with 1.25 nM Rad51. These measurements were complicated by the fact that 85%–90% of molecules tested would compact to the surface of the flow cell. However, this is to be expected, as experiments at 0.5 pN of force primarily resulted in DNA compaction via optical trapping (Fig. 1E). For activity measurements, we focused on molecules that did not compact to the surface. We chose these molecules because they are the most likely to define single remodeling events associated with an isolated PSC. Our selection criteria were based on analyzing data until the bead extension dropped below the initial extension. At this point, we no longer used data from these molecules even if they returned to baseline or above.

We observed a concentration-dependent increase in the change in extension between 500 and 125 pM PSC (1.28 vs. 1.1 µm, *P* = 0.0007) (Supplemental Fig. S3B). There was also an increase in the half-life of events formed at higher PSC concentrations (∼10 sec longer) (Supplemental Fig. S3C). However, no difference in extensions per second was observed, suggesting that the rate was unaffected by concentration (Supplemental Fig. S3D). From these measurements, we conclude that the stability and size of remodeled DNA were affected by the concentration of the PSC. These differences could reflect differences between a 1D translocation-based search and a 3D diffusion-based search, as PSCs with longer lifetimes are more likely to move along the DNA processively.

By performing activity measurements at −60 turns, we observed the interaction between Rad51–ssDNA filaments and the donor DNA in the absence of Rad54. Interestingly, Rad51–ssDNA extended DNA by ∼0.44 µm. This was 0.82 µm smaller than the corresponding PSC measurements (Fig. 4C). Additionally, the change in extensions per second and the half-lives of the events were significantly shorter than the PSC (Fig. 4D,E). We also evaluated the contribution of ATP hydrolysis by forming PSCs with AMPPNP instead of ATP. Under these conditions, the extensions, extensions per second, and half-lives of the events were all the same as those of Rad51–ssDNA alone (Fig. 4C–E). These data suggest that ATP hydrolysis by Rad54 is required for additional DNA remodeling and for stabilizing the PSC on the DNA.

To better understand the independent contributions of Rad54 and Rad51 in this experiment, we used a mutant form of Rad51 with three amino acid substitutions: R188A, K361A, and K371A. These substitutions disrupt the DNA-binding site II within Rad51 and are referred to as Rad51-II3A (Cloud et al. 2012). This site is conserved in all recombinases, and mutations in these residues fail in the homology search (Renkawitz et al. 2013, 2014) and strand exchange (Cloud et al. 2012). We reasoned that Rad51-II3A would fail to interact with the partially opened donor dsDNA, and PSCs with Rad51-II3A would have smaller extensions or extension rates or shorter lifetimes. It should be noted that this mutant retains significant affinity for both ssDNA and dsDNA but lacks the ability to initiate strand exchange or complete the homology search (Cloud et al. 2012; Renkawitz et al. 2014). At 500 pM PSC with Rad51-II3A, there was a loss of ∼0.37 µm from the extension events, comparable with the length of the Rad51–ssDNA filaments (Fig. 4C; Supplemental Fig. S3B). At lower concentrations, this loss was less (∼0.27 µm) (Supplemental Fig. S3B). However, this could be due to reduced Rad54-mediated DNA extension under these conditions. There was also a difference observed between the PSC and the PSC with Rad51-II3A in the extensions per second (Fig. 4D; Supplemental Fig. S3D), a difference of ∼0.153 µm/sec, suggesting that Rad51–ssDNA contacts the donor DNA during probing events. No difference in half-life was observed between PSCs and PSCs with Rad51-II3A. The absence of Rad51–ssDNA extension indicates that the filaments are no longer able to probe the donor DNA for homology. From this, we conclude that Rad54 can increase the binding and stability of Rad51 filaments in the absence of sequence homology by underwinding the DNA.

We asked whether Rad51 contributed to the formation of the larger loops observed via optical trapping or whether it simply remodeled DNA in an isolated region. If Rad51 contributed to the formation of larger loops, then the Rad51-II3A mutant would show a reduction in loop size or loop extrusion rate. This was not the case, and we observed that the mean loop size was comparable with that of WT at 0.5 pN of force (Fig. 4F). There was also no significant difference in the amount of initial DNA isolation or the number of intermediates formed, suggesting that there was no loss in the structural integrity of the Rad51–ssDNA filament (Supplemental Fig. S4A,B). We also did not observe a reduction in the translocation rate for PSCs with Rad51-II3A at 5 pN of force (Fig. 4G). There was a small increase in the force required to separate intermediate contacts that formed during compaction upon re-extension (Fig. 4H). From this, we conclude that there is no significant impact of Rad51 binding II on the formation of large, isolated loops. Instead, it suggests that Rad51-binding site II primarily affects the extension of the donor DNA and is likely required for the initiation of strand exchange or the initial stability of the paired product.

### The twisting of DNA is reduced at higher forces

**Figure 5.**
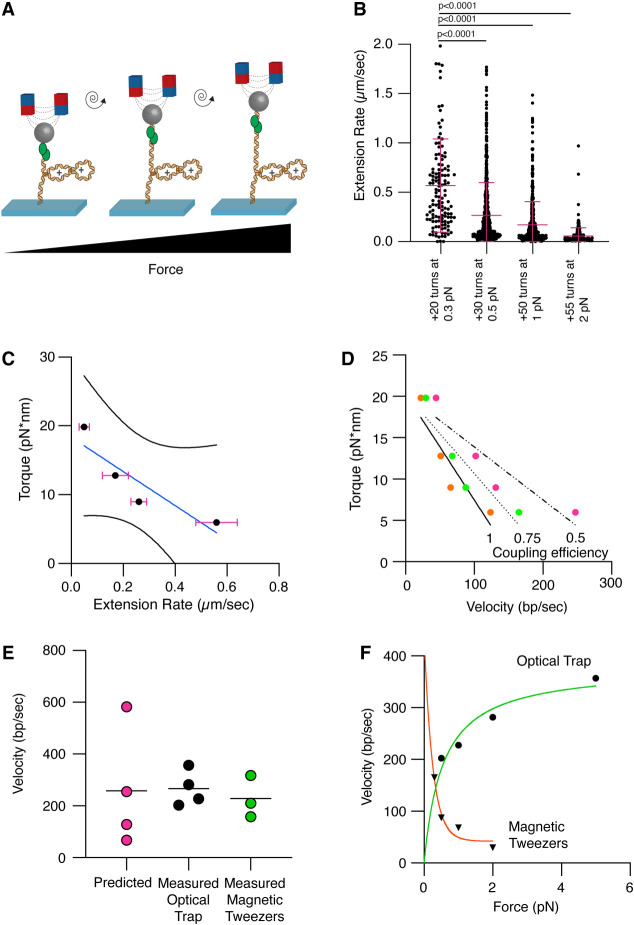
The PSC fails to turn DNA at high force. (*A*) Schematic of a magnetic tweezer experiment with DNA held at different levels of force. (*B*) A graph representing the DNA extension rate for the PSC at 0.3 pN (*N* = 129), 0.5 pN (*N* = 587), 1.0 pN (*N* = 477), and 5.0 pN (*N* = 274) of force. The bar represents the mean, and the error bars indicate the standard deviation of the data. Significant differences between the data sets were determined using an unpaired *t*-test, and the *P*-values are shown in the graph. (*C*) Torque plot generated by graphing the mean extension rate for each measurement at different forces and plotting it against the torque on the DNA. The error bars represent the 95% confidence interval of the data, and the blue line represents a linear fit. The dashed lines represent the 95% confidence interval of the linear fit. (*D*) Torque plots using coupling efficiencies of 1 (orange), 0.75 (green), and 0.5 (pink) to estimate the loop extrusion velocity in base pairs per second. All three of these estimates were extrapolated using the data set shown in *B* and *C*. (*E*) Comparison of the modeled loop extrusion rate (pink) at twofold, threefold, fivefold, and 10-fold stimulation (10-fold is the top point); the measured optical trap rates at 0.5, 1.0, 2.0, and 5.0 pN of force (black; 5 pN is the top data point); and the loop extrusion measured by magnetic tweezers (green) using 1.0, 0.75, and 0.5 coupling efficiency (0.5-fold coupling efficiency is the top data point). (*F*) Comparison of velocity measurements made at different forces for optical trapping experiments (green) and magnetic tweezer experiments for a coupling efficiency of 0.75 (orange). The lines represent the fit for a rectangular hyperbola (green) and single-phase exponential decay (orange).

In a cell, torsional forces acting on DNA within topologically isolated regions exert torque on the DNA. By monitoring PSC activity on underwound DNA, we were able to understand how an assisting torque affected loop extrusion activity and stability. We next used magnetic tweezers to perform experiments on overwound DNA, allowing us to monitor PSC activity under resisting torque. In contrast to underwound DNA, overwound DNA can still undergo buckling transitions at forces >1 pN. After buckling, the torque on the DNA is fixed, and additional twists added to the system are stored as writhe (Strick et al. 1998; Forth et al. 2008; Brutzer et al. 2010; Gao et al. 2021). By performing experiments at 0.3, 0.5, 1.0, and 2.0 pN of force, more torque is added to the DNA prior to buckling, and the DNA is more resistant to the addition of turns (Fig. 5A). Importantly, this alters the DNA's stiffness, making the condition more comparable with stretching the DNA on the optical trap but in a constrained system.

As expected, Rad51–ssDNA and the PSC were less active on overwound DNA, and this resulted in reduced extension size, extension rate, and lifetime of binding to the DNA (Supplemental Fig. S5A–C). Increasing force on the bead resulted in a systematic reduction in PSC activity for extension, extension rate, and half-life (Fig. 5B; Supplemental Fig. S5D,E). Using the change in extension rate, we generated a torque plot for the PSC by converting force to torque using a previously established conversion (Fig. 5C; Gao et al. 2021). By using a linear fit of the data (*r*^2^ = 0.89), we then extrapolated a stall torque for the PSC of 20 pN/nm. This value is comparable with those of RNA polymerase and the replication machinery (Ma et al. 2013, 2019; Jia et al. 2025; Qian et al. 2026) and would be sufficient to disrupt nucleosomes (Sheinin et al. 2013). It should be noted that the extrapolation of the stall torque using a linear fit is a simplification of a relationship that is not entirely linear. Therefore, the estimate of 20 pN/nm may underestimate the precise value of the stall torque.

The *X*-intercept of our torque plot represents the “no torque” velocity. To determine whether this rate was comparable with the velocity measured in our model and on the optical trap, we converted the extension rate to base pairs per second. In this experimental setup, directly converting to base pairs per second requires knowledge of the motor's efficiency in adding turns to the DNA during translocation. This variable, called the coupling efficiency, is difficult to measure directly. Furthermore, it may change depending on the DNA's stiffness. Therefore, we generated torque plots that reflected different levels of coupling. For example, at a 1:1 coupling efficiency, one turn is added to the superhelical axis of DNA for every 10.5 bp traveled. This assumption estimates a “no torque” activity of 158 bp/sec (Fig. 5D). At lower coupling efficiency, this value was 211 (0.75) and 317 (0.5) bp/sec, respectively. These values were comparable with those observed by optical trapping and with those from our PSC-mediated loop extrusion model (Fig. 5E). Finally, we compared the trend lines for the optical trapping measurements and magnetic tweezer experiments. We observed an inverse relationship between translocation rate and force: Higher forces led to faster linear translocation, while lower forces led to higher loop extrusion rates (Fig. 5F). This observation indicates that compaction results from twisting DNA at low force and that linear movement dominates as DNA becomes more difficult to twist.

At the end of each experiment, the magnet was rotated from −70 to 70 and back. This could identify any changes to the hat curve that occurred during the reaction. For Rad51–ssDNA alone, a translational shift in the hat curve was observed, similar to that observed for RecA–ssDNA in the presence of AMPPNP (Supplemental Fig. S6A; van der Heijden et al. 2008). Interestingly, when the same measurement was made with the full PSC, the negative side of the hat curve became flatter and did not undergo a buckling transition (Supplemental Fig. S6B). Similar observations were made for the PSC with Rad51-II3A (Supplemental Fig. S6C), with the exception that these hat curves were shorter. The slope of the hat curve from −70 to −20 turns was used to make a comparative assessment. The value was substantially different between Rad51–ssDNA, the PSC, and the PSC with Rad51-II3A (Supplemental Fig. S6D). These data are consistent with donor DNA that is prone to melt instead of buckle, and this effect was dependent on Rad54 concentration in the PSC (Supplemental Fig. S6D). Rad54 alone did not cause this outcome (Supplemental Fig. S6D). This suggests that DNA bound by a PSC is predisposed to melt upon the addition of superhelical stress or torsion. In this scenario, as additional turns are added to the DNA, it will undergo a melting transition and not form a plectoneme. One interpretation is that Rad54 predisposes the donor DNA to melt when Rad51 recognizes complete homology, representing a novel mechanism by which D-loops might be stabilized.

### Mutants defective in DNA compaction fail to form D-loops in vivo

**Figure 6.**
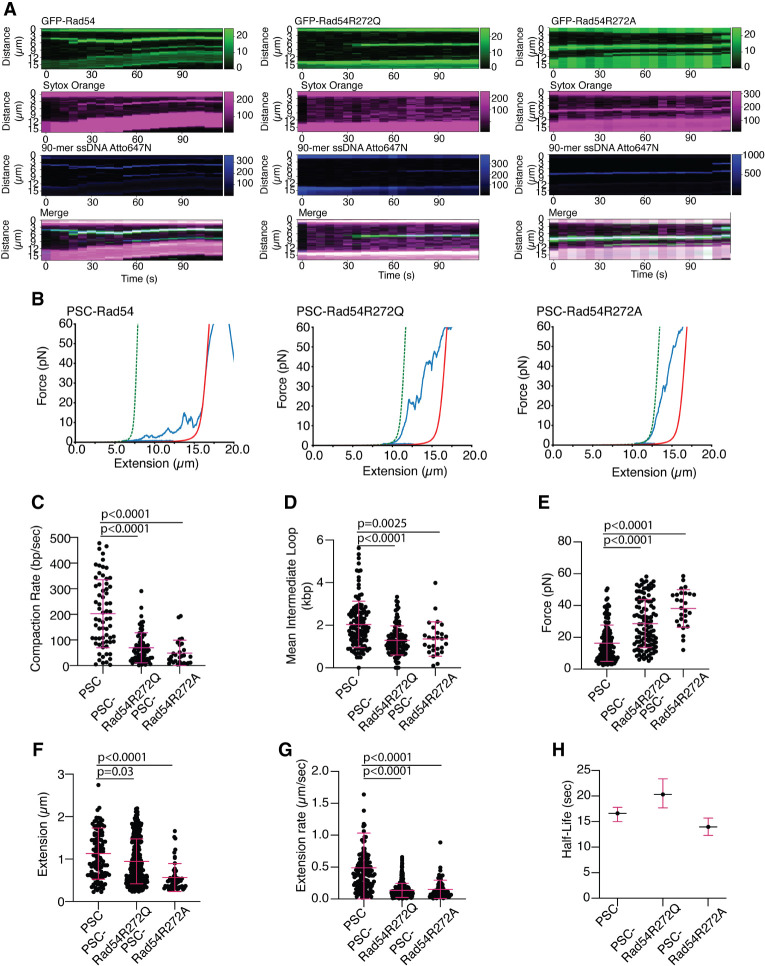
Defects in processive translocation alter loop dynamics. (*A*) Representative kymographs for the PSC (*left*), the PSC with Rad54 R272Q (*middle*), and the PSC with Rad54 R272A (*right*). (*B*) Representative postcompaction FE curves for the PSC (*left*), the PSC with Rad54 R272Q (*middle*), and the PSC with Rad54 R272A (*right*). The blue line represents the measured forces, and the red line represents the theoretical force extension curve for the DNA. The green dashed line represents the initial point of deviation from the theoretical FE curve. (*C*) A graph comparing the loop extrusion rate for the PSC (*N* = 66), the PSC with Rad54 R272Q (*N* = 70), and the PSC with Rad54 R272A (*N* = 28) at 0.5 pN. The crossbar represents the mean of the data, and the error bars indicate the standard deviation. The PSC data are reproduced from Figure 1F. (*D*) A graph that represents the mean intermediate loop size for the PSC (*N* = 130), the PSC with Rad54 R272Q (*N* = 111), and the PSC with Rad54 R272Q (*N* = 27). The crossbar represents the mean of the data, and the error bars indicate the standard deviation. The PSC data are reproduced from Figure 2. (*E*) A dot plot represents the force required to disrupt interactions in compacted DNA structures for the PSC (*N* = 130), the PSC with Rad54 R272Q (*N* = 111), and the PSC with Rad54 R272Q (*N* = 27). The crossbar represents the mean of the data, and the error bars indicate the standard deviation. The PSC data are reproduced from Figure 2. (*F*) Graph representing extension for the MT data of 125 pM PSC (*N* = 120), the PSC with Rad54 R272Q (*N* = 362), and the PSC with Rad54 R272A (*N* = 54). The crossbar represents the mean, and the error bars indicate the standard deviation. The PSC data are reproduced from Supplemental Figure S3. (*G*) Graph representing the extension per second for 125 pM PSC (*N* = 148), 125 pM PSC with Rad54 R272Q (*N* = 402), and 125 pM PSC with Rad54 R272A (*N* = 70). The line represents the mean, and the error bars indicate the standard deviation of the experiment. (*H*) Graph representing the extension half-life for the PSC (*N* = 120), the PSC with Rad54 R272Q (*N* = 362), and the PSC with Rad54 R272A (*N* = 54). The dot represents the half-life, and the error bars indicate the 95% confidence of the fit. Statistical significance was determined using an unpaired *t*-test.

We have previously characterized mutations in Rad54 that result in defects in processive movement of the PSC along donor DNA. These mutant versions of Rad54 are defective for homology search in vitro (Sridalla et al. 2024). Defects stem from insufficient ATP hydrolysis required for translocation. We reasoned that these Rad54 mutants (Rad54 R272Q and Rad54 R272A) would be defective in generating loops and extending the DNA. We initially measured the activity of PSCs with these mutants by optical trapping experiments (Fig. 6A,B). As expected, there was a 3.2.-fold reduction in the loop extrusion rate for the PSC with Rad54 R272Q and a 5.1-fold reduction for Rad54 R272A (Fig. 6C). The mutant forms of Rad54 in the PSC also exhibited an ∼800 bp reduction in mean loop size (Fig. 6D). There was also a significant reduction in the size of the initially constrained loops in both Rad54 R272Q and Rad54 R272A (Supplemental Fig. S7A). In the Rad54 R272Q group, this was not due to a change in the number of contact points, suggesting no structural defect in the PSC. However, the Rad54 R272A mutant displayed a reduced number of contacts with the DNA, suggesting a further defect in the structural integrity of the PSC (Supplemental Fig. S7B). Re-extension of the DNA after compaction required 1.75 times the mean force to disrupt intermediate contacts in Rad54 R272Q and 2.3 times the mean force to disrupt intermediate contacts in Rad54 R272A (Fig. 6E). From this, we conclude that PSCs with Rad54 mutants are defective in loop compaction, likely due to reduced processive activity.

We next evaluated how these substitutions affected the change in DNA extension at −60 turns. These experiments were performed at 125 pM PSC. Under these conditions, the WT PSC extended the DNA by ∼1.13 µm (Fig. 6F; Supplemental Fig. S7C–E). The PSC Rad54 R272Q mutant extended DNA by ∼0.94 nm(∼83% of the WT value). The PSC Rad54 R272A mutant extended DNA by 0.56 µm, representing a twofold reduction (Fig. 6F). The DNA extension rate per second was significantly reduced, with the Rad54 R272Q and Rad54 R272A mutants both showing a 3.2-fold defect (Fig. 6G). The extension half-life was slightly longer than that of the WT for the Rad54 R272Q mutant, but there was no difference for the Rad54 R272A mutant, suggesting that the half-life of these events was comparable with that of WT (Fig. 6H). While these mutations are defective in all aspects of the PSC activity, the most severe defects are associated with DNA compaction and the rate of DNA extension (Sridalla et al. 2024). Together, these data suggest that the ability to act processively is critical for PSC function.

**Figure 7.**
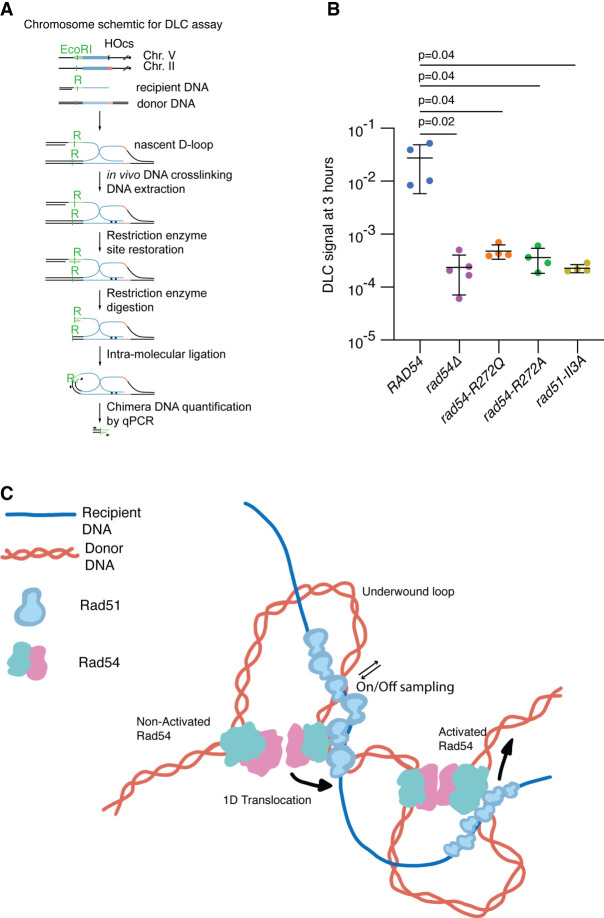
Failure in the processive search limits D-loop formation in vivo. (*A*) Diagram illustrating the D-loop capture experiment used in *S. cerevisiae* cells to determine the efficiency of nascent D-loop capture in cells. (*B*) A graph representing the nascent D-loop capture efficiency for *RAD54*, *rad54*Δ, *rad54 R272Q*, *rad54 R272A*, and *rad51-II3A.* The crossbar represents the mean of the data, and the error bars indicate the standard error of at least three independent experiments. (*C*) Diagram illustrating the model for cooperative homology search by Rad51 and Rad54.

To determine whether these mutants were defective in target search in vivo, we used a reporter assay in *S. cerevisiae* that measures the amount of D-loop capture during early strand exchange (Piazza et al. 2019; Reitz et al. 2022). The experiment is based on an HO nuclease site at a specific location on chromosome V, with a homologous region on chromosome II (Fig. 7A). The HO nuclease can be induced with galactose, and the broken recipient DNA can undergo a homology search and D-loop formation. Nascent D-loops can be detected using psoralen cross-linking to trap them in cells. Psoralen is a DNA intercalating agent activated by UV whose activity depends on DNA topology. One caveat to this approach is that psoralen binding is higher when DNA is underwound, but binding can cause the DNA to become slightly overwound. Therefore, if small unstable joints exist, they could be disrupted. Therefore, captured products likely reflect nascent D-loops that have been partially stabilized.

We monitored nascent D-loop capture at 3 h and found that in WT strains, D-loops were captured at an efficiency of 3.3 ± 0.06 × 10^−2^ (Fig. 7B). In contrast, *rad54*Δ had a capture efficiency of 1.7 ± 0.096 × 10^−4^ (Fig. 7B), representing a 200-fold reduction compared with that of WT. The *rad54 R272Q* and *rad54 R272A* strains both captured D-loops 80-fold less efficiently than WT (Fig. 7B). This was surprising because these mutations are less severe than those in *rad54*Δ in methyl methanesulfonate (MMS) sensitivity assays, and longer ssDNA fragments can form D-loops in vitro (Sridalla et al. 2024). We expected both mutants to retain partial function; however, this was not the case. We also measured D-loop capture efficiency for the *rad51-II3A* mutant. This mutation results in a failed homology search in vivo (Renkawitz et al. 2013) and in a failure to remodel the donor DNA in our magnetic tweezer experiments. This mutant resulted in loss of D-loop capture in vivo (Fig. 7B) and highlights the requirement for Rad51 during the initial sequence selection during the homology search. Together, these experiments demonstrate that failure in initial nucleotide selection or in the processivity of the PSC leads to failure of the homology search in vivo.

## Discussion

The RecA family of recombinases forms filaments on ssDNA that can introduce forces onto duplex donor DNA in search of DNA sequence homology. In eukaryotes, this effort is enhanced through the recruitment of Rad54 to the PSC. Rad54 adds a translocation activity to the homology search complex, which can relax the superhelical structure of the donor DNA substrate and promote linear movement. These activities provide an efficient way to enhance Rad51–ssDNA filament binding to the donor duplex prior to or during homology recognition. Here, we demonstrated that the ATP-dependent motor protein Rad54 can catalyze Rad51–ssDNA binding in the absence of homology by providing assisting forces. The ability of Rad54 to regulate Rad51–ssDNA binding aids the search for homologous bases and is essential for sequence identification and D-loop formation in vivo.

### Optimizing translocation versus loop extrusion

DNA loops are common in biology and generally depend on the distance between two points of protein–DNA contact (Blumberg et al. 2005; Ding et al. 2014; Fogg et al. 2021). Loop formation can occur through direct capture of an existing loop or by pumping DNA into an isolated region (Maeshima and Iida 2021). Both mechanisms of loop formation depend on DNA stiffness, and our data show that Rad54, as part of the PSC, can generate loops via both mechanisms. Loop extrusion can further modify local DNA topology, provided the turns added to the DNA remain constrained. Our work provides direct evidence that Rad54 can constrain regions by creating multiple points of contact with the donor DNA, adding negative turns to the extruded loop.

The translocation activity of the PSC can occur as loop extrusion or linear movement. The distribution of these activities is regulated by the amount of force on the DNA and, by extension, DNA stiffness. Under physiological conditions, naked DNA is likely under low forces, and most forces generated in vivo likely result from accumulated torsional stress created by DNA motor activity (Ma et al. 2013). Torsional stress is regulated by chromatin structures and DNA supercoiling throughout the genome (Kaczmarczyk et al. 2020; Qian et al. 2026). This dynamic landscape may help the PSC balance linear movement and loop extrusion, both of which are necessary for the homology search and sequence identification.

The one-dimensional translocation-based activity during the homology search has come under scrutiny because of the potential impediment posed by nucleosomes and the relatively slow rate at which the PSC translocates (Crickard et al. 2020b; Sridalla et al. 2024). Both RecA and Rad51 filaments have been demonstrated to undergo one-dimensional and three-dimensional diffusion-based homology search mechanisms (Granéli et al. 2006; Forget and Kowalczykowski 2012; Ragunathan et al. 2012). Diffusion events, while the fastest way to search the genome, often result in low-energy collisions that do not produce stable binding, and multiple collision events are required to form a stable product. This creates a speed–stability paradox (Mirny et al. 2009). As noted, a homology search at a rate of 150–250 bp/sec would be inefficient for covering significant portions of the genome (Reitz et al. 2021). This makes it unlikely that the 1D translocation is the only means for search, and the true homology search is likely a combination of 1D translocation and diffusion within the genome. Incorporation of a translocation-based search method likely helps resolve the speed versus stability paradox by improving the efficiency of target selection and conversion to stable products once suitable homology has been identified.

The mechanism behind target selection and stable product formation is the controlled relaxation of the underlying donor DNA by loop extrusion. The presence of underwound loops in the PSC prior to primary sequence selection can couple initial sequence identification to stable product formation, thereby fully catalyzing sequence selection during the homology search (Fig. 7C). We imagine that these loops may form transiently during the homology search and become stable only when a homologous sequence is identified.

Our data show that Rad51–ssDNA filaments can contact and alter donor DNA in the absence of Rad54 and sequence homology. This interaction is stronger when DNA is underwound, consistent with previous experiments (Van Komen et al. 2000; Jaskelioff et al. 2003). Earlier work on chromatin substrates in vitro demonstrated that Rad51–ssDNA is sufficient to form short, unstable joint molecules but is unable to convert them to stable products. In nucleosome arrays, the linker DNA is underwound, while histone-bound DNA is slightly overwound (Naughton et al. 2013; Nikitina et al. 2017). These observations support the idea that DNA topology regulates sequence selection during the homology search. Nucleosome arrays will exhibit characteristics like the donor-bound PSC, with regions of negative helical density behind Rad54 and positive helical density ahead of Rad54. Our model is consistent with this earlier observation and suggests that dynamic regulation of DNA topology during the homology search may provide a kinetic mechanism for sequence identification and selection. Control of DNA topology can occur through nucleosome shuffling or through control of DNA secondary structure within isolated loops.

A second argument against a 1D translocation-based model is the presence of nucleosomes on the donor DNA. Rad54 is related to the ATPase subunits of ySwi/Snf (Snf2) and the RSC (Sth1), and previous reports have shown that these complexes can form DNA loops, which are likely related to the flexibility of the DNA. Like Rad54, these motors switch between loop extrusion and linear translocation. Unlike earlier studies, our work provides a direct comparison of these two activities. Rad54, in the context of its biological complex, the PSC, can generate and stabilize loops that are at least an order of magnitude larger than those of other remodeling enzymes, and these loops could contain multiple nucleosomes. The relevant quantity when addressing how the PSC deals with chromatin is the torque generated during translocation, and Rad54 produces torque comparable with that of other chromatin-remodeling enzymes.

The forces that govern nucleosome stability are related to interactions between the DNA and H2A/B (outer turn) and H3/H4 (inner turn). Each of these turns requires a different level of destabilizing force, which can lead to sliding or eviction (Sheinin et al. 2013). The 20 pN/nm torque produced by the PSC is sufficient to remodel nucleosomes and can lead to outer turn unwrapping ∼20% of the time (Sheinin et al. 2013), potentially leading to loss of the H2A/B dimer or the H3/H4 tetramer. Previous reports have measured encounters between individual nucleosomes and the PSC (Alexeev et al. 2003; Crickard et al. 2020b). These encounters result in nucleosome loss in ∼20% of cases where the nucleosome was remodeled, consistent with these values. It is unclear how Rad54 and the PSC may interact with nucleosome groups. Still, previous reports have suggested that homology can be found even within nucleosomal arrays (Zhang et al. 2007) and that Rad54 effectively repositions nucleosomes in vivo (Wolner and Peterson 2005). Future work will be needed to understand the limitations that nucleosome groups impose on PSC movement. However, a growing body of evidence suggests that nucleosomes will not create a significant impediment to PSC movement during the homology search, even in the presence of 1D translocation.

### Failures in the homology search

Mutations that disrupted ATP hydrolysis by Rad54 also failed to promote the homology search and nascent D-loop capture in *S. cerevisiae*. In vitro, these defects cause lower loop formation rates and smaller loop sizes. These outcomes reflect an enzyme that fails to turn over, resulting in a homology search complex that becomes stuck to the donor DNA or fails to convert unstable products into stable products. The outcomes of these experiments underscore the importance of Rad54 turnover during homology search, as it facilitates 1D translocation and regulates PSC stability. Interestingly, the Rad51-II3A mutant fails to contact the DNA in the presence of Rad54, suggesting that this mutant is defective in sampling the donor DNA and is likely unable to stabilize the 8–9 nt of homology required for primary sequence selection, the first step in strand exchange.

Recently, Rad54's participation in the homology search was measured in live cells. In baker's yeast, the deletion of Rad54 leads to the lengthening of Rad51 filaments (Liu et al. 2023), possibly due to the loss of donor DNA compaction, resulting in an apparent increase in filament size. In human cells, RAD54L was required to resolve RAD51 foci during the homology search. However, this mechanism is unclear. A cooperative interaction between human RAD54L and cohesin was also observed during the homology search (Friskes et al. 2025) and could occur by regulating the forces applied to the DNA. Further work will be needed to understand the direct biochemical relationship between the SMC protein cohesin and the PSC (Marin-Gonzalez et al. 2025; Teloni et al. 2025).

It remains to be seen whether RAD54L will make similar contributions during the homology search in human cells. Human RAD51 binds dsDNA more effectively in the context of the PSC, and additional factors, such as BRCA2 and RAD51AP, have evolved to regulate RAD51 binding to DNA (Pires et al. 2017; Selemenakis et al. 2022; Belan et al. 2023; Uhrig et al. 2024; Neal et al. 2025). While RAD54L is required for the resolution of RAD51 foci in human cells, this could be due to the removal of RAD51 filaments or modulation of PSC binding by donor DNA remodeling. Resolution of these possibilities will require the use of hypomorphic alleles specifically designed to separate these functions.

### Limitations of our study

A limitation of our study is that it is unclear how well conserved this mechanism might be between yeast and human Rad54. Although the proteins are 50% identical, differences in overall function may depend on context. Additionally, our experiments did not reveal the actual outcomes of sequence recognition and the formation of full D-loops. Finally, we did not measure PSC activity on chromatinized DNA, which would have provided better physiological context. Future work will be needed to address these three key points.

## Materials and methods

Yeast strains and plasmids used in this study are listed in Supplemental Tables S1 and S2.

### Protein purification

Rad54, Rad54 R272Q, Rad54 R272A, Rad51, and Rad51-IIA were purified as described previously (Crickard et al. 2020b). A more detailed description is in the Supplemental Material.

### Loop formation simulation

#### Model overview

Rad54 functions as a dimer during DNA translocation. Upon recruitment by Rad51, one of the Rad54 molecules becomes activated, resulting in an approximately fivefold increase in both ATP hydrolysis activity and DNA translocation velocity. Asymmetry in motor activity drives DNA loop extrusion. The loop formation rate (LFR) was modeled as proportional to the absolute difference in velocities between two Rad54 motors as follows: LFR = ∣*v*_1_ − *v*_2_∣, where *v*_1_ is the velocity of the unactivated motor, and *v*_2_ is the velocity of the activated motor.

#### Simulation procedure

The velocities *v*_1_ and *v*_2_ were sampled from two normal distributions, *v*_1_ ∼ *N*(*µ*, *σ*^2^) and *v*_2_ ∼ *N*(*A* × *µ*, *σ*^2^), where *µ* and *σ* describes the mean and standard deviation of the unactivated motor velocity, respectively, and *A* describes the activation fold after binding to Rad51. For a given condition (*µ*, *σ*, and *A*), we randomly sampled *v*_1_ and *v*_2_, discarded negative values, and then computed LFR using LFR = ∣*v*_1_ − *v*_2_∣. We repeated the process 1000 times and plotted the distribution of the calculated LFR.

### DNA template construction

The torsionally constrained DNA template was generated by adding an ∼500 bp multilabeled adapter at each end of a 12,667 bp DNA center, for a total length of ∼13,667 bp, as described previously (Le et al. 2023; Lee et al. 2023). A more detailed description is in the Supplemental Material.

### Magnetic tweezers

Experiments on the magnetic tweezers (MTs) were performed on a custom-built instrument (Le et al. 2023), allowing for bulk analysis of multiple DNA tethers under a constant force (Le et al. 2025). DNA oligos used in this study are listed in Supplemental Table S3. In each chamber, between 30 and 50 tethers remained constrained (TC) throughout the experiment and were affected by the rotation of the magnetic beads. Chambers for the MTs were prepared by nitrocellulose coating (1%–2% collodion in amyl acetate) on two microscope coverslips, forming a flow cell with the nitrocellulose surfaces facing inward. The surface of the chamber was then functionalized with antidigoxigenin (Vector Laboratories MB-7000), passivated with 1.25 mg/mL β-casein (Sigma C6905), incubated with 1–2 pM 12.7 kb λ-DNA template, and incubated with streptavidin-coated paramagnetic beads (Invitrogen 65601). To ensure no free beads remained in the solution, a buffer exchange was done to start all chambers in HR buffer [30 mM Tris-OAc at pH 7.5, 10 mM Mg(OAc)_2_, 50 mM NaCl, 1.5 mg/mL β-casein, 1 mM DTT]. Before introducing Rad54 to the sample chamber, DNA tethers were assayed to determine torsional constraint and to provide a baseline for unadulterated tether behavior. The tethers were twisted via rotation of a magnet until overwound and underwound, leading to the generation of a buckling curve. Tethers were also left in an overwound or underwound state for 2 min compared with tethers with bound protein. To load protein, the force in the chamber was increased to 6 pN while the protein flowed in for 2 min, after which the tethers were overwound/underwound, and the force dropped to the experimental force. A monitor was engaged for 10 min, after which the initial buckling (hat) curve was regenerated for further analysis. Changes to the hat curve were analyzed by measuring the slope of the negative side of the curve. This was compared with DNA without proteins.

### MT data analysis

Activity traces were collected over 10 min periods. Active traces were determined by using the fluctuation of the torsionally constrained DNA alone as a baseline. Changes in extension were identified by determining local maximum/minimum within an extension event. A change in extension was considered an event when it exceeded 3 standard deviations (SD) from the baseline, as determined solely from the DNA. Positive rates were determined by identifying the slope leading to a local maximum. Negative rates were determined by identifying the slope following a local maximum. Slopes were linear between maxima/minima. Extension data were smoothed by applying a 5 sec sliding window. The mean extension for a given DNA template was determined by fitting to a Gaussian distribution. The lifetime of each extension event was determined by setting a threshold of the mean extension of naked DNA under torsion and analyzing traces that extended +3 SD from the baseline. Any events that crossed this threshold lasting >2.5 sec were considered active traces. The lifetime of events was determined by the time between points that were >3 SD from the mean. These lifetimes fit an exponential decay curve. The code used for all MT analysis is available on GitHub.

### Torque plots and base pair per second rate estimation

The amount of torque felt by the DNA was determined using the following conversion: torque = 12.7825 × force^0.63447^ pN/nm. This conversion was determined from previously published measurements (Gao et al. 2021). Velocity in base pairs per second was estimated by first using the slope of the buckling transition from the hat curve for each individual tether to convert observed changes in micrometers per second to turns per second. At 10.5 bp/turn, consistent with B-form DNA, this was further converted to rates of base pairs per second using the coupling efficiency. The mean turns per second were then multiplied by the estimated coupling efficiency to generate the plots in Figure 5, D–F. It should be noted that these represent estimates, and other approaches could be used to provide more precise measurements.

### Lumicks confocal microscopy with optical trapping

All fluorescent experiments were conducted on a Lumicks C-trap instrument, allowing for the combination of an optical dual trap with confocal imaging microscopy. Excitation lasers at 488, 532, and 647 nm allowed for the excitation of GFP (Rad54), dsDNA (Sytox orange), and ssDNA (Atto-647N), respectively. Before experiments, the five channel laminar flow cell (model C1) was passivated using 0.5 mg/mL β-casein (Sigma C6905) in a standard running buffer (PBS; 1.5 mM sodium azide, 0.5 mM EDTA). To form DNA tethers, 5 µm streptavidin-coated polystyrene beads (0.004% [w/v] [Lumicks], diluted in running buffer) were trapped and moved briefly into the biotinylated λ-DNA channel (8 pg/µL [Lumicks], diluted in running buffer). After tether formation, the traps were moved to the HR buffer in channel 3 and stretched to ensure single tether formation. All protein was loaded into channel 4 in HR buffer and 1 mM ATP. For catching beads, forming tethers, and loading protein, the flow was kept at a constant 0.2 ± 0.05 bar. The trapping power was set to 7.5% during data collection, leading to a trap stiffness of ∼0.07 pN/nm. Kymographs were collected with 0.5 msec pixel dwell time for each 100 nm pixel. The kymograph frame rate alternated between 2 frames per second (0.5 msec exposure) and 10 frames per second (0.25 msec exposure), depending on experimental needs. Unless dictated otherwise, kymographs were collected using staggered excitation lasers, where each laser was on for 1 sec and off for 2 sec. For conditions where the 532 nm lasers were not used, excitation alternated between 488 and 647 nm lasers in 1 sec increments. These were temporally offset to prevent bleed-through of the channels.

### Lumicks confocal microscope with optical trap (C-trap) experimental protocols

The experimental protocol for Lumicks experiments was separated into three automated scripts. All experiments were followed by overstretching the DNA at a constant rate. The elastic parameters of dsDNA used to generate the theoretical force extension curve were obtained from Wang et al. (1997). These parameters were also used to convert from raw data into force, extension, and DNA base pairs.

### Force clamp experiments

Captured DNA tethers were moved into channel 4 at a high (16 µm) extension. Protein was loaded via 60 sec of flow. After protein loading, a force clamp was entered at 0.5, 1, 2, or 5 pN, and a kymograph was generated using 488/532/647 nm lasers at 2 frames per second (FPS).

### Force extension measurements

Captured DNA tethers were moved into channel 4 at varying extensions (6, 8, 10, 12, and 14 µm). Protein was loaded via 30 sec of flow. After loading protein, the tether was moved to a 12.5 µm extension, equivalent to 0.5 pN of force on naked DNA. Kymographs were generated using 488/647 nm lasers at 2 frames per second (FPS). Force extension curves were also collected during the experiment.

### Lumicks data analysis

Raw data exported from Lumicks Bluelake as .h5 files were processed in Spyder using Python 3.10 and custom-made software. Kymographs were generated, and all measurements were taken from the intensity data included in these kymographs. Particle tracking was done by manually selecting bound proteins. The resulting particles were used to measure velocities, binding lifetimes, and fluorescent intensities. Translocation velocities were calculated by measuring the distance changed over unit time. Compaction measurements, the degree of compaction, and the compaction rate were analyzed by changes in the distances between the two beads as measured by the extension curve. Re-extension of the compacted DNA was performed at a defined rate, and measurements were performed by analyzing the force extension curve. The size of defined loops was determined from the size of the DNA when a loop disruption resulted in a return to a theoretical re-extension curve. Each loop disruption was included for further analysis. Analysis of force measurements was performed by analyzing the force extension curve during translocation. Maximum force intensities during translocation were used to generate mean force output.

### DLC assay

DLC assay was performed as described previously (Piazza et al. 2019; Reitz et al. 2022). DNA oligos used in this study are listed in Supplemental Table S3. Yeast cells were grown overnight in 5 mL of yeast extract peptone (YP) medium supplemented with 2% dextrose and 4% adenine sulfate. The second day, the culture was diluted by 10-fold in 5 mL of YP plus 3% glycerol, 2% lactate, and 4% adenine sulfate and grown for ∼8 h. Next, the culture was inoculated into 100 mL of YP plus 3% glycerol, 2% lactate, and 4% adenine sulfate medium with an initial OD_600_ ≈ 0.006 and grown for 16 h. A 5× psoralen stock solution (0.5 mg/mL trioxsalen in 200 proof ethanol) was made in a 50 mL aluminum foil-covered tube and dissolved on a shaker overnight at room temperature with gentle rocking. The next day, the culture had an OD_600_ of 0.3–0.8, and 7.5 OD_600_ of cells was collected as time 0 control and centrifuged at 2246*g* for 5 min at 4°C. The cell pellets were resuspended in 1× psoralen buffer. The 1× psoralen buffer was prepared by diluting 5× psoralen in 200 proof ethanol before collecting cells. The resuspended cells were plated in a 60 mm Petri dish, which was placed 2–3 cm below a UV light source with the lip removed on top of a prechilled metal block. The cell samples were exposed under the UV light for 10 min with gentle shaking to cross-link DNA. The cells were transferred to a 15 mL Falcon tube. The Petri dish was rinsed with TE1 solution (50 mM Tris-Cl at pH 8.0, 50 mM EDTA at pH 8.0), and the TE1 buffer was poured together with the cells. The cells were then centrifuged again at 2246*g* for 5 min at 4°C, and the pellets were saved at −20°C. Galactose was added to the culture to a final concentration of 2% to induce DSBs. Cells were collected at 3 h after galactose treatment.

The cell pellets were thawed on ice, resuspended in spheroplasting buffer (0.4 M sorbitol, 0.4 M KCl, 40 mM sodium phosphate buffer at pH 7.2, 0.5 mM MgCl_2_), and transferred to a 1.7 mL microcentrifuge tube. The cells were then spheroplasted in zymolyase solution (2% glucose, 50 mM Tris-Cl at pH 7.5, 5 mg/mL zymolyase 100T) for 20 min at 30°C. The cells were washed three times with spheroplasting buffer at 2500*g* and three times with restriction enzyme (RE) buffer [50 mM KOAc, 20 mM Tris-OAc, 10 mM Mg(OAc)_2_, 1 mg/mL BSA] at 16,000*g*. The pellets were resuspended with 1.4× RE buffer without or with a hybridization oligo to restore the EcoRI restriction sites, flash-frozen using dry ice, and then stored at −80°C.

The DNA was solubilized by incubating the cells with 0.1% SDS for 13 min at 65°C. The SDS was quenched with 1% Triton X-100. The DNA was digested by 20 U of EcoRI for 1 h at 37°C. The restriction enzyme was deactivated by incubating the DNA with 1.5% SDS for 10 min at 55°C. The cells were put back on ice, and the SDS was quenched by the addition of 6% Triton X-100. Ligation buffer (50 mM Tris-HCl at pH 8.0, 10 mM MgCl_2_, 10 mM DTT, 2.5 µg/mL BSA, 1 mM ATP at pH 8.0, 8 U of T4 DNA ligase) was added to perform ligation reaction for 1.5 h at 16°C. Proteinase K (25 µμg/mL) was added to digest the enzymes for 30 min at 65°C. DNA was extracted by adding phenol:chloroform:isoamyl alcohol and vortexing. The upper water phase was transferred to a tube, incubated with 0.10 vol of NaOAc and an equal volume of isopropanol for 30 min at room temperature, and centrifuged at 21,130*g* for 10 min at 4°C to obtain DNA precipitation. The DNA pellets were dried at 37°C and dissolved by incubating with 1× TE buffer (10 mM Tris-HCl at pH 8.0, 1 mM EDTA) for 1 h at 37°C. The DNA was used as a qPCR template. DLC chimera content was calculated by $\mathrm{DLC}\;\mathrm{content}={\left(\mathrm{DLC}\;\mathrm{amplification}\;\mathrm{efficiency}\right)}^{-Cp_{\mathrm{DLC}}}$, and the intramolecular ligation product content was calculated by ligationcontent=(ligationamplification ${\mathrm{efficiency})}^{-Cp_{\mathrm{ligation}}}$. The final DLC signal was calculated by

$$\frac{\mathrm{DLC}\;\mathrm{content}}{\mathrm{ligation}\;\mathrm{content}}.$$

### Data availability

Data, including kymographs, have been submitted to the Mendeley repository (https://data.mendeley.com/preview/w8scwmdnyh?a=09ab573b-edf5-499f-b96f-fb57f8336c4c). This repository will be published upon acceptance of this manuscript. All analysis codes have been uploaded to GitHub (https://github.coecis.cornell.edu/dlm345/Woodhouse-GD-Paper). Additionally, all strains are available on request from J.B.C. (jbc287@cornell.edu).

### Competing interest statement

The authors declare no competing interests.

## Supplemental Material

Supplement 1

## References

[GAD353376WOOC1] Alexeev A, Mazin A, Kowalczykowski SC. 2003. Rad54 protein possesses chromatin-remodeling activity stimulated by the Rad51-ssDNA nucleoprotein filament. Nat Struct Biol 10: 182–186. 10.1038/nsb901 12577053

[GAD353376WOOC2] Alexiadis V, Lusser A, Kadonaga JT. 2004. A conserved N-terminal motif in Rad54 is important for chromatin remodeling and homologous strand pairing. J Biol Chem 279: 27824–27829. 10.1074/jbc.M402648200 15105430

[GAD353376WOOC3] Amitani I, Baskin RJ, Kowalczykowski SC. 2006. Visualization of Rad54, a chromatin remodeling protein, translocating on single DNA molecules. Mol Cell 23: 143–148. 10.1016/j.molcel.2006.05.009 16818238

[GAD353376WOOC4] Baumann CG, Bloomfield VA, Smith SB, Bustamante C, Wang MD, Block SM. 2000. Stretching of single collapsed DNA molecules. Biophys J 78: 1965–1978. 10.1016/S0006-3495(00)76744-0 10733975 PMC1300789

[GAD353376WOOC5] Belan O, Greenhough L, Kuhlen L, Anand R, Kaczmarczyk A, Gruszka DT, Yardimci H, Zhang X, Rueda DS, West SC, 2023. Visualization of direct and diffusion-assisted RAD51 nucleation by full-length human BRCA2 protein. Mol Cell 83: 2925–2940.e8. 10.1016/j.molcel.2023.06.031 37499663 PMC7615647

[GAD353376WOOC6] Bell JC, Kowalczykowski SC. 2016. RecA: regulation and mechanism of a molecular search engine. Trends Biochem Sci 41: 491–507. 10.1016/j.tibs.2016.04.002 27156117 PMC4892382

[GAD353376WOOC7] Blumberg S, Tkachenko AV, Meiners J-C. 2005. Disruption of protein-mediated DNA looping by tension in the substrate DNA. Biophys J 88: 1692–1701. 10.1529/biophysj.104.054486 15653717 PMC1305226

[GAD353376WOOC8] Brutzer H, Luzzietti N, Klaue D, Seidel R. 2010. Energetics at the DNA supercoiling transition. Biophys J 98: 1267–1276. 10.1016/j.bpj.2009.12.4292 20371326 PMC2849096

[GAD353376WOOC9] Ceballos SJ, Heyer WD. 2011. Functions of the Snf2/Swi2 family Rad54 motor protein in homologous recombination. Biochim Biophys Acta 1809: 509–523. 10.1016/j.bbagrm.2011.06.006 21704205 PMC3171615

[GAD353376WOOC10] Cloud V, Chan YL, Grubb J, Budke B, Bishop DK. 2012. Rad51 is an accessory factor for Dmc1-mediated joint molecule formation during meiosis. Science 337: 1222–1225. 10.1126/science.1219379 22955832 PMC4056682

[GAD353376WOOC11] Crickard JB, Kwon Y, Sung P, Greene EC. 2020a. Rad54 and Rdh54 occupy spatially and functionally distinct sites within the Rad51–ssDNA presynaptic complex. EMBO J 39: e105705. 10.15252/embj.2020105705 32790929 PMC7560196

[GAD353376WOOC12] Crickard JB, Moevus CJ, Kwon Y, Sung P, Greene EC. 2020b. Rad54 drives ATP hydrolysis-dependent DNA sequence alignment during homologous recombination. Cell 181: 1380–1394.e18. 10.1016/j.cell.2020.04.056 32502392 PMC7418177

[GAD353376WOOC13] Danilowicz C, Peacock-Villada A, Vlassakis J, Facon A, Feinstein E, Kleckner N, Prentiss M. 2014. The differential extension in dsDNA bound to Rad51 filaments may play important roles in homology recognition and strand exchange. Nucleic Acids Res 42: 526–533. 10.1093/nar/gkt867 24084082 PMC3874182

[GAD353376WOOC14] Danilowicz C, Fu J, Prentiss M. 2024. Insight into RecA-mediated repair of double strand breaks is provided by probing how contiguous heterology affects recombination. J Biol Chem 300: 107887. 10.1016/j.jbc.2024.107887 39395797 PMC11570958

[GAD353376WOOC15] De Vlaminck I, van Loenhout MT, Zweifel L, den Blanken J, Hooning K, Hage S, Kerssemakers J, Dekker C. 2012. Mechanism of homology recognition in DNA recombination from dual-molecule experiments. Mol Cell 46: 616–624. 10.1016/j.molcel.2012.03.029 22560720

[GAD353376WOOC16] Ding Y, Manzo C, Fulcrand G, Leng F, Dunlap D, Finzi L. 2014. DNA supercoiling: a regulatory signal for the λ repressor. Proc Natl Acad Sci 111: 15402–15407. 10.1073/pnas.1320644111 25319264 PMC4217475

[GAD353376WOOC17] Dumont A, Mendiboure N, Savocco J, Anani L, Moreau P, Thierry A, Modolo L, Jost D, Piazza A. 2024. Mechanism of homology search expansion during recombinational DNA break repair in *Saccharomyces cerevisiae*. Mol Cell 84: 3237–3253.e6. 10.1016/j.molcel.2024.08.003 39178861

[GAD353376WOOC18] Flaus A, Martin DM, Barton GJ, Owen-Hughes T. 2006. Identification of multiple distinct Snf2 subfamilies with conserved structural motifs. Nucleic Acids Res 34: 2887–2905. 10.1093/nar/gkl295 16738128 PMC1474054

[GAD353376WOOC19] Fogg JM, Judge AK, Stricker E, Chan HL, Zechiedrich L. 2021. Supercoiling and looping promote DNA base accessibility and coordination among distant sites. Nat Commun 12: 5683. 10.1038/s41467-021-25936-2 34584096 PMC8478907

[GAD353376WOOC20] Forget AL, Kowalczykowski SC. 2012. Single-molecule imaging of DNA pairing by RecA reveals a three-dimensional homology search. Nature 482: 423–427. 10.1038/nature10782 22318518 PMC3288143

[GAD353376WOOC21] Forth S, Deufel C, Sheinin MY, Daniels B, Sethna JP, Wang MD. 2008. Abrupt buckling transition observed during the plectoneme formation of individual DNA molecules. Phys Rev Lett 100: 148301. 10.1103/PhysRevLett.100.148301 18518075 PMC3019760

[GAD353376WOOC22] Friskes A, Snoek M, Oldenkamp R, van den Broek B, Nahidiazar L, Koob L, Dick AE, Mertz M, Harkes R, Rowland BD, 2025. Visualizing homology search in living cells. bioRxiv 10.1101/2025.03.01.640932 PMC13563089 42725434

[GAD353376WOOC23] Gao X, Hong Y, Ye F, Inman JT, Wang MD. 2021. Torsional stiffness of extended and plectonemic DNA. Phys Rev Lett 127: 028101. 10.1103/PhysRevLett.127.028101 34296898 PMC9007542

[GAD353376WOOC24] Granéli A, Yeykal CC, Robertson RB, Greene EC. 2006. Long-distance lateral diffusion of human Rad51 on double-stranded DNA. Proc Natl Acad Sci 103: 1221–1226. 10.1073/pnas.0508366103 16432240 PMC1345706

[GAD353376WOOC25] Greene EC. 2016. DNA sequence alignment during homologous recombination. J Biol Chem 291: 11572–11580. 10.1074/jbc.R116.724807 27129270 PMC4882428

[GAD353376WOOC26] Haber JE. 2018. DNA repair: the search for homology. Bioessays 40: e1700229. 10.1002/bies.201700229 29603285 PMC6238635

[GAD353376WOOC27] Havas K, Flaus A, Phelan M, Kingston R, Wade PA, Lilley DMJ, Owen-Hughes T. 2000. Generation of superhelical torsion by ATP-dependent chromatin remodeling activities. Cell 103: 1133–1142. 10.1016/S0092-8674(00)00215-4 11163188

[GAD353376WOOC28] Hopfner KP, Gerhold CB, Lakomek K, Wollmann P. 2012. Swi2/Snf2 remodelers: hybrid views on hybrid molecular machines. Curr Opin Struct Biol 22: 225–233. 10.1016/j.sbi.2012.02.007 22445226 PMC3323801

[GAD353376WOOC29] Jasin M, Rothstein R. 2013. Repair of strand breaks by homologous recombination. Cold Spring Harb Perspect Biol 5: a012740. 10.1101/cshperspect.a012740 24097900 PMC3809576

[GAD353376WOOC30] Jaskelioff M, Van Komen S, Krebs JE, Sung P, Peterson CL. 2003. Rad54p is a chromatin remodeling enzyme required for heteroduplex DNA joint formation with chromatin. J Biol Chem 278: 9212–9218. 10.1074/jbc.M211545200 12514177

[GAD353376WOOC31] Jia X, Gao X, Zhang S, Inman JT, Hong Y, Singh A, Patel S, Wang MD. 2025. Torsion is a dynamic regulator of DNA replication stalling and reactivation. Nat Commun 16: 10543. 10.1038/s41467-025-65567-5 41298417 PMC12658067

[GAD353376WOOC32] Kaczmarczyk A, Meng H, Ordu O, van Noort J, Dekker NH. 2020. Chromatin fibers stabilize nucleosomes under torsional stress. Nat Commun 11: 126. 10.1038/s41467-019-13891-y 31913285 PMC6949304

[GAD353376WOOC33] Kowalczykowski SC. 2015. An overview of the molecular mechanisms of recombinational DNA repair. Cold Spring Harb Perspect Biol 7: a016410. 10.1101/cshperspect.a016410 26525148 PMC4632670

[GAD353376WOOC34] Le TT, Gao X, Park SH, Lee J, Inman JT, Lee JH, Killian JL, Badman RP, Berger JM, Wang MD. 2019. Synergistic coordination of chromatin torsional mechanics and topoisomerase activity. Cell 179: 619–631.e15. 10.1016/j.cell.2019.09.034 31626768 PMC6899335

[GAD353376WOOC35] Le TT, Wu M, Lee JH, Bhatt N, Inman JT, Berger JM, Wang MD. 2023. Etoposide promotes DNA loop trapping and barrier formation by topoisomerase II. Nat Chem Biol 19: 641–650. 10.1038/s41589-022-01235-9 36717711 PMC10154222

[GAD353376WOOC36] Le TT, Gao X, Ha Park S, Lee J, Inman JT, Wang MD. 2025. Protocol for effective surface passivation for single-molecule studies of chromatin and topoisomerase II. STAR Protoc 6: 103500. 10.1016/j.xpro.2024.103500 39693223 PMC11719840

[GAD353376WOOC37] Lee JY, Terakawa T, Qi Z, Steinfeld JB, Redding S, Kwon Y, Gaines WA, Zhao W, Sung P, Greene EC. 2015. DNA recombination. Base triplet stepping by the Rad51/RecA family of recombinases. Science 349: 977–981. 10.1126/science.aab2666 26315438 PMC4580133

[GAD353376WOOC38] Lee J, Wu M, Inman JT, Singh G, Park SH, Lee JH, Fulbright RM, Hong Y, Jeong J, Berger JM, 2023. Chromatinization modulates topoisomerase II processivity. Nat Commun 14: 6844. 10.1038/s41467-023-42600-z 37891161 PMC10611788

[GAD353376WOOC39] Liu S, Miné-Hattab J, Villemeur M, Guerois R, Pinholt HD, Mirny LA, Taddei A. 2023. In vivo tracking of functionally tagged Rad51 unveils a robust strategy of homology search. Nat Struct Mol Biol 30: 1582–1591. 10.1038/s41594-023-01065-w 37605042

[GAD353376WOOC40] Ma J, Bai L, Wang MD. 2013. Transcription under torsion. Science 340: 1580–1583. 10.1126/science.1235441 23812716 PMC5657242

[GAD353376WOOC41] Ma J, Tan C, Gao X, Fulbright RM Jr, Roberts JW, Wang MD. 2019. Transcription factor regulation of RNA polymerase's torque generation capacity. Proc Natl Acad Sci 116: 2583–2588. 10.1073/pnas.1807031116 30635423 PMC6377492

[GAD353376WOOC42] Maeshima K, Iida S. 2021. The loopy world of cohesin. eLife 10: e71585. 10.7554/eLife.71585 34309512 PMC8313229

[GAD353376WOOC43] Marin-Gonzalez A, Rybczynski AT, Nilavar NM, Nguyen D, Li AG, Karwacki-Neisius V, Zou RS, Avilés-Vázquez FJ, Kanemaki MT, Scully R, 2025. Cohesin drives chromatin scanning during the RAD51-mediated homology search. Science 390: eadw1928. 10.1126/science.adw1928 41343630 PMC12701822

[GAD353376WOOC44] Mazin AV, Bornarth CJ, Solinger JA, Heyer WD, Kowalczykowski SC. 2000. Rad54 protein is targeted to pairing loci by the Rad51 nucleoprotein filament. Mol Cell 6: 583–592. 10.1016/S1097-2765(00)00057-5 11030338

[GAD353376WOOC45] Mirny L, Slutsky M, Wunderlich Z, Tafvizi A, Leith J, Kosmrlj A. 2009. How a protein searches for its site on DNA: the mechanism of facilitated diffusion. J Phys A Math Theor 42: 434013. 10.1088/1751-8113/42/43/434013

[GAD353376WOOC46] Naughton C, Avlonitis N, Corless S, Prendergast JG, Mati IK, Eijk PP, Cockroft SL, Bradley M, Ylstra B, Gilbert N. 2013. Transcription forms and remodels supercoiling domains unfolding large-scale chromatin structures. Nat Struct Mol Biol 20: 387–395. 10.1038/nsmb.2509 23416946 PMC3689368

[GAD353376WOOC47] Neal FE, Li W, Uhrig ME, Katz JN, Syed S, Sharma N, Dutta A, Burma S, Hromas R, Mazin AV, 2025. Distinct roles of the two BRCA2 DNA-binding domains in DNA damage repair and replication fork preservation. Cell Rep 44: 115654. 10.1016/j.celrep.2025.115654 40323719 PMC12129652

[GAD353376WOOC48] Nikitina T, Norouzi D, Grigoryev SA, Zhurkin VB. 2017. DNA topology in chromatin is defined by nucleosome spacing. Sci Adv 3: e1700957. 10.1126/sciadv.1700957 29098179 PMC5659657

[GAD353376WOOC49] Petukhova G, Stratton S, Sung P. 1998. Catalysis of homologous DNA pairing by yeast Rad51 and Rad54 proteins. Nature 393: 91–94. 10.1038/30037 9590697

[GAD353376WOOC50] Petukhova G, Van Komen S, Vergano S, Klein H, Sung P. 1999. Yeast Rad54 promotes Rad51-dependent homologous DNA pairing via ATP hydrolysis-driven change in DNA double helix conformation. J Biol Chem 274: 29453–29462. 10.1074/jbc.274.41.29453 10506208

[GAD353376WOOC51] Piazza A, Shah SS, Wright WD, Gore SK, Koszul R, Heyer WD. 2019. Dynamic processing of displacement loops during recombinational DNA repair. Mol Cell 73: 1255–1266.e4. 10.1016/j.molcel.2019.01.005 30737186 PMC6532985

[GAD353376WOOC52] Piazza A, Bordelet H, Dumont A, Thierry A, Savocco J, Girard F, Koszul R. 2021. Cohesin regulates homology search during recombinational DNA repair. Nat Cell Biol 23: 1176–1186. 10.1038/s41556-021-00783-x 34750581

[GAD353376WOOC53] Pires E, Sung P, Wiese C. 2017. Role of RAD51AP1 in homologous recombination DNA repair and carcinogenesis. DNA Repair 59: 76–81. 10.1016/j.dnarep.2017.09.008 28963981 PMC5643253

[GAD353376WOOC54] Prasad TK, Robertson RB, Visnapuu ML, Chi P, Sung P, Greene EC. 2007. A DNA-translocating Snf2 molecular motor: *Saccharomyces cerevisiae* Rdh54 displays processive translocation and extrudes DNA loops. J Mol Biol 369: 940–953. 10.1016/j.jmb.2007.04.005 17467735 PMC2705995

[GAD353376WOOC55] Qi Z, Redding S, Lee JY, Gibb B, Kwon Y, Niu H, Gaines WA, Sung P, Greene EC. 2015. DNA sequence alignment by microhomology sampling during homologous recombination. Cell 160: 856–869. 10.1016/j.cell.2015.01.029 25684365 PMC4344887

[GAD353376WOOC56] Qian J, Lubkowska L, Zhang S, Tan C, Hong Y, Jia X, Fulbright RM, Inman JT, Kay TM, Jeong J, 2026. Chromatin buffers torsional stress during transcription. Science 391: eadv0134. 10.1126/science.adv013441411413

[GAD353376WOOC57] Ragunathan K, Liu C, Ha T. 2012. RecA filament sliding on DNA facilitates homology search. eLife 1: e00067. 10.7554/eLife.00067 23240082 PMC3510455

[GAD353376WOOC58] Raschle M, Van Komen S, Chi P, Ellenberger T, Sung P. 2004. Multiple interactions with the Rad51 recombinase govern the homologous recombination function of Rad54. J Biol Chem 279: 51973–51980. 10.1074/jbc.M410101200 15465810

[GAD353376WOOC59] Reitz D, Chan YL, Bishop DK. 2021. How strand exchange protein function benefits from ATP hydrolysis. Curr Opin Genet Dev 71: 120–128. 10.1016/j.gde.2021.06.016 34343922 PMC8671154

[GAD353376WOOC60] Reitz D, Savocco J, Piazza A, Heyer WD. 2022. Detection of homologous recombination intermediates via proximity ligation and quantitative PCR in *Saccharomyces cerevisiae*. J Vis Exp 10.3791/64240 PMC10205173 36155960

[GAD353376WOOC61] Renkawitz J, Lademann CA, Kalocsay M, Jentsch S. 2013. Monitoring homology search during DNA double-strand break repair in vivo. Mol Cell 50: 261–272. 10.1016/j.molcel.2013.02.020 23523370

[GAD353376WOOC62] Renkawitz J, Lademann CA, Jentsch S. 2014. Mechanisms and principles of homology search during recombination. Nat Rev Mol Cell Biol 15: 369–383. 10.1038/nrm3805 24824069

[GAD353376WOOC63] Ristic D, Wyman C, Paulusma C, Kanaar R. 2001. The architecture of the human Rad54–DNA complex provides evidence for protein translocation along DNA. Proc Natl Acad Sci 98: 8454–8460. 10.1073/pnas.151056798 11459989 PMC37457

[GAD353376WOOC64] Sanchez H, Kertokalio A, van Rossum-Fikkert S, Kanaar R, Wyman C. 2013. Combined optical and topographic imaging reveals different arrangements of human RAD54 with presynaptic and postsynaptic RAD51–DNA filaments. Proc Natl Acad Sci 110: 11385–11390. 10.1073/pnas.1306467110 23801766 PMC3710881

[GAD353376WOOC65] San Filippo J, Sung P, Klein H. 2008. Mechanism of eukaryotic homologous recombination. Annu Rev Biochem 77: 229–257. 10.1146/annurev.biochem.77.061306.125255 18275380

[GAD353376WOOC66] Selemenakis P, Sharma N, Uhrig ME, Katz J, Kwon Y, Sung P, Wiese C. 2022. RAD51AP1 and RAD54L can underpin two distinct RAD51-dependent routes of DNA damage repair via homologous recombination. Front Cell Dev Biol 10: 866601. 10.3389/fcell.2022.866601 35652094 PMC9149245

[GAD353376WOOC67] Sheinin MY, Forth S, Marko JF, Wang MD. 2011. Underwound DNA under tension: structure, elasticity, and sequence-dependent behaviors. Phys Rev Lett 107: 108102. 10.1103/PhysRevLett.107.108102 21981534 PMC3201814

[GAD353376WOOC68] Sheinin MY, Li M, Soltani M, Luger K, Wang MD. 2013. Torque modulates nucleosome stability and facilitates H2A/H2B dimer loss. Nat Commun 4: 2579. 10.1038/ncomms3579 24113677 PMC3848035

[GAD353376WOOC69] Sinha M, Peterson CL. 2008. A Rad51 presynaptic filament is sufficient to capture nucleosomal homology during recombinational repair of a DNA double-strand break. Mol Cell 30: 803–810. 10.1016/j.molcel.2008.04.015 18570881 PMC4461863

[GAD353376WOOC70] Solinger JA, Lutz G, Sugiyama T, Kowalczykowski SC, Heyer WD. 2001. Rad54 protein stimulates heteroduplex DNA formation in the synaptic phase of DNA strand exchange via specific interactions with the presynaptic Rad51 nucleoprotein filament. J Mol Biol 307: 1207–1221. 10.1006/jmbi.2001.4555 11292336

[GAD353376WOOC71] Sridalla K, Woodhouse MV, Hu J, Scheer J, Ferlez B, Crickard JB. 2024. The translocation activity of Rad54 reduces crossover outcomes during homologous recombination. Nucleic Acids Res 52: 7031–7048. 10.1093/nar/gkae474 38828785 PMC11229335

[GAD353376WOOC72] Strick TR, Allemand JF, Bensimon D, Croquette V. 1998. Behavior of supercoiled DNA. Biophys J 74: 2016–2028. 10.1016/S0006-3495(98)77908-1 9545060 PMC1299542

[GAD353376WOOC73] Strick T, Allemand J-F, Croquette V, Bensimon D. 2000. Twisting and stretching single DNA molecules. Prog Biophys Mol Biol 74: 115–140. 10.1016/S0079-6107(00)00018-3 11106809

[GAD353376WOOC74] Sugawara N, Wang X, Haber JE. 2003. In vivo roles of Rad52, Rad54, and Rad55 proteins in Rad51-mediated recombination. Mol Cell 12: 209–219. 10.1016/S1097-2765(03)00269-7 12887906

[GAD353376WOOC75] Tan TL, Kanaar R, Wyman C. 2003. Rad54, a jack of all trades in homologous recombination. DNA Repair 2: 787–794. 10.1016/S1568-7864(03)00070-3 12826279

[GAD353376WOOC76] Tavares EM, Wright WD, Heyer WD, Le Cam E, Dupaigne P. 2019. In vitro role of Rad54 in Rad51–ssDNA filament-dependent homology search and synaptic complexes formation. Nat Commun 10: 4058. 10.1038/s41467-019-12082-z 31492866 PMC6731316

[GAD353376WOOC77] Teloni F, Takacs Z, Mitter M, Langer CCH, Prlesi I, Steinacker TL, Reuter VP, Mylarshchikov D, Gerlich DW. 2025. Cohesin guides homology search during DNA repair using loops and sister chromatid linkages. Science 390: eadw0566. 10.1126/science.adw0566 41343653

[GAD353376WOOC78] Thomä NH, Czyzewski BK, Alexeev AA, Mazin AV, Kowalczykowski SC, Pavletich NP. 2005. Structure of the SWI2/SNF2 chromatin-remodeling domain of eukaryotic Rad54. Nat Struct Mol Biol 12: 350–356. 10.1038/nsmb919 15806108

[GAD353376WOOC79] Uhrig ME, Sharma N, Maxwell P, Gomez J, Selemenakis P, Mazin AV, Wiese C. 2024. Disparate requirements for RAD54L in replication fork reversal. Nucleic Acids Res 52: 12390–12404. 10.1093/nar/gkae828 39315725 PMC11551752

[GAD353376WOOC80] van der Heijden T, Modesti M, Hage S, Kanaar R, Wyman C, Dekker C. 2008. Homologous recombination in real time: DNA strand exchange by RecA. Mol Cell 30: 530–538. 10.1016/j.molcel.2008.03.010 18498754

[GAD353376WOOC81] Van Komen S, Petukhova G, Sigurdsson S, Stratton S, Sung P. 2000. Superhelicity-driven homologous DNA pairing by yeast recombination factors Rad51 and Rad54. Mol Cell 6: 563–572. 10.1016/S1097-2765(00)00055-1 11030336

[GAD353376WOOC82] van Mameren J, Modesti M, Kanaar R, Wyman C, Peterman EJ, Wuite GJ. 2009. Counting RAD51 proteins disassembling from nucleoprotein filaments under tension. Nature 457: 745–748. 10.1038/nature07581 19060884 PMC3871861

[GAD353376WOOC83] Vlassakis J, Feinstein E, Yang D, Tilloy A, Weiller D, Kates-Harbeck J, Coljee V, Prentiss M. 2013. Tension on dsDNA bound to ssDNA–RecA filaments may play an important role in driving efficient and accurate homology recognition and strand exchange. Phys Rev E 87: 032702. 10.1103/PhysRevE.87.032702 PMC4973255 27499708

[GAD353376WOOC84] Wang MD, Yin H, Landick R, Gelles J, Block SM. 1997. Stretching DNA with optical tweezers. Biophys J 72: 1335–1346. 10.1016/S0006-3495(97)78780-0 9138579 PMC1184516

[GAD353376WOOC85] Wiktor J, Gynnå AH, Leroy P, Larsson J, Coceano G, Testa I, Elf J. 2021. RecA finds homologous DNA by reduced dimensionality search. Nature 597: 426–429. 10.1038/s41586-021-03877-6 34471288 PMC8443446

[GAD353376WOOC86] Wolner B, Peterson CL. 2005. ATP-dependent and ATP-independent roles for the Rad54 chromatin remodeling enzyme during recombinational repair of a DNA double strand break. J Biol Chem 280: 10855–10860. 10.1074/jbc.M414388200 15653683

[GAD353376WOOC87] Wolner B, van Komen S, Sung P, Peterson CL. 2003. Recruitment of the recombinational repair machinery to a DNA double-strand break in yeast. Mol Cell 12: 221–232. 10.1016/S1097-2765(03)00242-9 12887907

[GAD353376WOOC88] Wright WD, Heyer WD. 2014. Rad54 functions as a heteroduplex DNA pump modulated by its DNA substrates and Rad51 during D loop formation. Mol Cell 53: 420–432. 10.1016/j.molcel.2013.12.027 24486020 PMC4059524

[GAD353376WOOC89] Xu J, Zhao L, Xu Y, Zhao W, Sung P, Wang HW. 2017. Cryo-EM structures of human RAD51 recombinase filaments during catalysis of DNA-strand exchange. Nat Struct Mol Biol 24: 40–46. 10.1038/nsmb.3336 27941862 PMC5471492

[GAD353376WOOC90] Xu W, Dunlap D, Finzi L. 2021. Energetics of twisted DNA topologies. Biophys J 120: 3242–3252. 10.1016/j.bpj.2021.05.002 33974883 PMC8391063

[GAD353376WOOC91] Yang H, Pavletich NP. 2021. Insights into homology search from cryo-EM structures of RecA–DNA recombination intermediates. Curr Opin Genet Dev 71: 188–194. 10.1016/j.gde.2021.09.002 34592688 PMC8671187

[GAD353376WOOC92] Yang H, Zhou C, Dhar A, Pavletich NP. 2020. Mechanism of strand exchange from RecA–DNA synaptic and D-loop structures. Nature 586: 801–806. 10.1038/s41586-020-2820-9 33057191 PMC8366275

[GAD353376WOOC93] Zhang Y, Smith CL, Saha A, Grill SW, Mihardja S, Smith SB, Cairns BR, Peterson CL, Bustamante C. 2006. DNA translocation and loop formation mechanism of chromatin remodeling by SWI/SNF and RSC. Mol Cell 24: 559–568. 10.1016/j.molcel.2006.10.025 17188033 PMC9034902

[GAD353376WOOC94] Zhang Z, Fan HY, Goldman JA, Kingston RE. 2007. Homology-driven chromatin remodeling by human RAD54. Nat Struct Mol Biol 14: 397–405. 10.1038/nsmb1223 17417655

