# Supplementary material for: The eukaryotic homology search complex distorts donor DNA structure to probe for homology
Source: Genes Dev. 2026 Jun 1;40(11-12):832–51. doi: 10.1101/gad.353376.125 (PMC13119058; doi:10.1101/gad.353376.125)
Supplement: Supplement 1 [file Supplemental__Data.pdf]

Supplemental Information:

Supplemental Tables S1, S2, S3
S1,S2, and S3 tables are related to methods

Supplemental Methods

Supplemental Figures S1-7
S1 is related to Figures 1 and 2
S2 and S3 are related to Figure 4
S4 is related to Figure 5
S5 and S7 are related to Figure 6
S6 is related to Figure 4

Supplementary Table S1 Related to Methods
Strains used in the study

Strains	Genotype	Source or reference
WDHY5511	*ura3::A-HOcs, lys2::A, trp1::GAL-HO-hphMX, his3D200, can1-100, leu2-3,112, ade2-1, RAD5.*	Piazza et al, *Methods in Enzymology*, 2018
JBC0423 (WDHY5511)	*rad54Δ::KANMX*	This study
JBC0281(WDHY5511)	*RAD54-KANMX*	This study
JBC0283(WDHY5511)	*rad54 R272Q-KANMX*	This study
JBC0487(WDHY5511)	*rad54 R272A-KANMX*	This study
JBC0534(WDHY5511)	*RAD54-KANMX, rad51-II3A-LEU2*	This study
Protease deficient yeast strain	*MAT alpha leu2 trp1 ura3–52 prb1–1122 his3::pGAL1/10*	Crickard et al, *Cell*, 2020

Supplementary Table S2 Related to Methods
Plasmid in the study

Backbone	Construction	Source
pRS305	*RAD54-KANMX*	This study
pRS305	*rad54-R272A-KANMX*	This study
pRS305	*rad54-R272Q-KANMX*	This study
pYES	*GST-GFP-RAD54*	Crickard et al EMBO 2018
pYES	*GST-GFP-RAD54 R272Q*	Sridalla et al Nucleic Acid Research 2024
pYES	*GST-GFP-RAD54 R272A*	Sridalla et al Nucleic Acid Research 2024
pET11C	*6xHis-SUMO-yRad51*	Crickard et al Cell 2020
pET11C	*6xHis-SUMO-yRad51II3A*	This Study
pNRFTC (pMDW111)	For torsionally constrained DNA	Le et al Cell 2019

Supplementary Table S3 Related to Methods
Oligos used in the study

Name	Sequence 5' to 3'	Purpose
90-mer DNA	Atto647N-GATGTTCTGCTGGATATGCACTTTTCCGGGCTGACGTACACCGTGCTCAGCCTGTTTTTCAGCGATCCGGATATGCATCCGCTGGATTTC	Single Molecule imaging
pNFRTC_BssSI_F	CAGTCACGAGGTTGTAAAACG	To make Biotin labeled adapters (Lee et al Nat Comms 2023)
pNFRTC_R	ACGCCAAGCTTCCACATC	To make Biotin and digoxygenin labeled adapters (Lee et al Nat Comms 2023)
pNFRTC_Ava1_F	GGGTAACGCTCGGGTTTTCC	To make digoxygenin labeled adapters (Lee et al Nat Comms 2023)
olWDH1760	CAGCGGGCTTGCAGAAGTTG	To amplify genomic DNA at *ARG4*
olWDH1761	GGCCAATTAGTTCACCAAGACG	To amplify genomic DNA at *ARG4*

olWDH1766	GTTTCAGCTTTCCGCAACAG	To quantify DSB induction
olWDH1767	GGCGAGGTATTGGATAGTTCC	To quantify DSB induction
olWDH1762	ACTTCGAATTTCGGCACTTC	To quantify intramolecular ligation efficiency of *Eco*RI-derived fragments
olWDH1763	CGATGAAACGTTAAGTGACCAC	To quantify intramolecular ligation efficiency of *Eco*RI-derived fragments
olWDH1764	AGAGCGGTCAGTAGCAATCC	To amplify at the upstream of DSB
olWDH1765	CACACGCGAAAAACCGCC	To amplify at the upstream of the donor DNA, used with olWDH1764 to quantify DLC signal
olWDH2019	CTTTAACCGGACGCTCGA	To quantify psoralen crosslinking efficiency
olWDH2020	TTGAGTTTATTGCTGCCGTC	To quantify psoralen crosslinking efficiency
olWDH1768	AGGAGCACAGACTTAGATTGG	Used with olWDH1764 to measure *Eco*RI recognition site restoration
olWDH1770	CGAAATCATCTTCGGTTAAATCCAAAACGGCAGAAGCCTGAATGAAACATATGAACCAATTGGAGGACGTCAATGAATTCTGGGGATCCATTGCATTTTT	To restore *Eco*RI site

Supplemental Methods

Protein purification:

In brief, a protease-deficient yeast strain was transformed with GFP-GST-Rad54, GFP-GST-Rad54 R272Q, or GFP-GST-Rad54 R272A on 2 2-micron plasmids under the control of the Gal1/10 promoter. Cells were grown in Yeast Nitrogen Base (–URA) plus 3% glycerol and 2% lactic acid. When the cells reached an OD_{600} of 1.5, expression was induced by adding 2% galactose for 6 hours. Cells were harvested and stored at –80°C.

Cell pellets were resuspended in Rad54 resuspension buffer (30 mM Tris–HCl [pH 7.5], 1 M NaCl, 1 mM EDTA, 10% glycerol, 10 mM BME (β-mercaptoethanol), protease inhibitor cocktail (Roche Cat. No. 05892953001), and 2 mM PMSF. Cells were disrupted by manual bead beating, and the lysate was clarified by centrifugation at 26,500xg for 1 hour. The lysate was fractionated by ammonium sulfate (AS) precipitation. AS was gradually added with mixing to a final concentration of 20% followed by centrifugation at 10,000 x g for 10 minutes. The supernatant was discarded, and the AS concentration was raised to 50% followed by centrifugation at 10,000 x g for 10 min. The protein pellet was resuspended in PBS (phosphate buffered saline) plus 1M NaCl and 10 mM BME. The resulting re-suspended protein was then bound to pre-equilibrated GST resin in batch for 1 hour at 4°C. The GST resin was washed 2x with PBS plus 1000 mM NaCl, and 2x with PBS plus 500 mM NaCl. The protein was eluted in 20 mM glutathione in PBS plus 500 mM NaCl. The peak fractions were pooled and then applied to a Sephacryl S–300 High Resolution gel filtration column (GE Healthcare, Cat. No. 17–0599–10) pre–equilibrated with Rad54 SEC buffer (30 mM Tris–HCl [pH 7.5], 500 mM NaCl, 1 mM EDTA, 10% glycerol, and

10 mM BME. The peak was pooled and dialyzed against Rad54 SEC buffer plus 50% glycerol and stored at –80°C in single-use aliquots.

6xHis–SUMO–Rad51 or 6xHis-SUMO-Rad51II3A was transformed into *E. coli* BL21 (DE3) Rosetta2 cells and grown to an OD_{600} of 0.4–0.6 at 37°C . Expression was induced by addition of 0.5 mM IPTG for 3 hours at 37°C. Cells were harvested and stored at –80°C. Cells were lysed by freeze–thaw in Cell Lysis Buffer (CLB:30 mM Tris–HCl [pH 8.0], 1 M NaCl, 10% glycerol, 10 mM imidazole, 5 mM BME, and protease inhibitor cocktail (Roche Cat. No. 05892953001)). Crude lysates were sonicated for 6 pulses of 30 seconds on and 2 minutes off, then clarified by centrifugation at 26,500 x g. The extract was precipitated with 50% AS and centrifuged at 26,500 x g for 10 minutes. The Pellet was resuspended in CLB and bound to 1 mL of pre–equilibrated Ni–NTA resin for 1 hour with rotation at 4 ˚C. The resin was washed 3x with CLB and eluted in CLB+200 mM imidazole. The protein was mixed with 400 units of the SUMO protease Ulp1 and dialyzed overnight at 4°C into Rad51 buffer (30 mM Tris–HCl [pH 8.0], 150 mM NaCl, 1 mM EDTA, 10% glycerol, 10 mM imidazole). The 6xHis–SUMO tag and SUMO protease were removed by passing the dialyzed proteins over a second 1 mL Ni–NTA column. The purified Rad51 was then stored at –80°C in single use aliquots.

Yeast Strain Construction

The initial yeast strain used in this study was a kind gift from Wolf Heyer. The *rad54*Δ strains were generated by gene knockout using a KanMX cassette. The *rad54R272Q*/A strain was generated by using gene replacement with a PCR product from a *pRS305-rad54R272Q/A-KanMX* plasmid. WT *RAD54* was similarly replaced to generate *RAD54-KanMX*, which was used as the

WT. In these constructs, the KanMX marker is 125 bp downstream of the stop codon in the Rad54 gene. The *rad51-II3A* mutant was constructed in a similar manner except a LEU2 selection was used to make the strain.

Magnetic Tweezers Template Construction:

The center segment was PCR amplified from λ-DNA (NEB, N3011S), then double digested with AvaI (NEB, R0152S) and BssSI-v2 (NEB, R0680S) to produce the unique overhangs for ligation. To make the 500-bp multi-biotin-labeled and multidigoxigenin-labeled adapters, we performed PCR amplification from plasmid pNFRTC (pMDW111) with either 24% of dATP replaced by biotin-14-dATP or 24% of dTTP replaced by digoxigenin-11-dUTP, followed by restriction enzyme digestion with BssSI and AvaI, respectively. The ~500-bp multi-labeled adapters with unique overhangs were ligated to the 12,688 bp center segment. The template ligation product was gel-purified, and aliquots were stored at -20 °C.

Supplemental Figures:

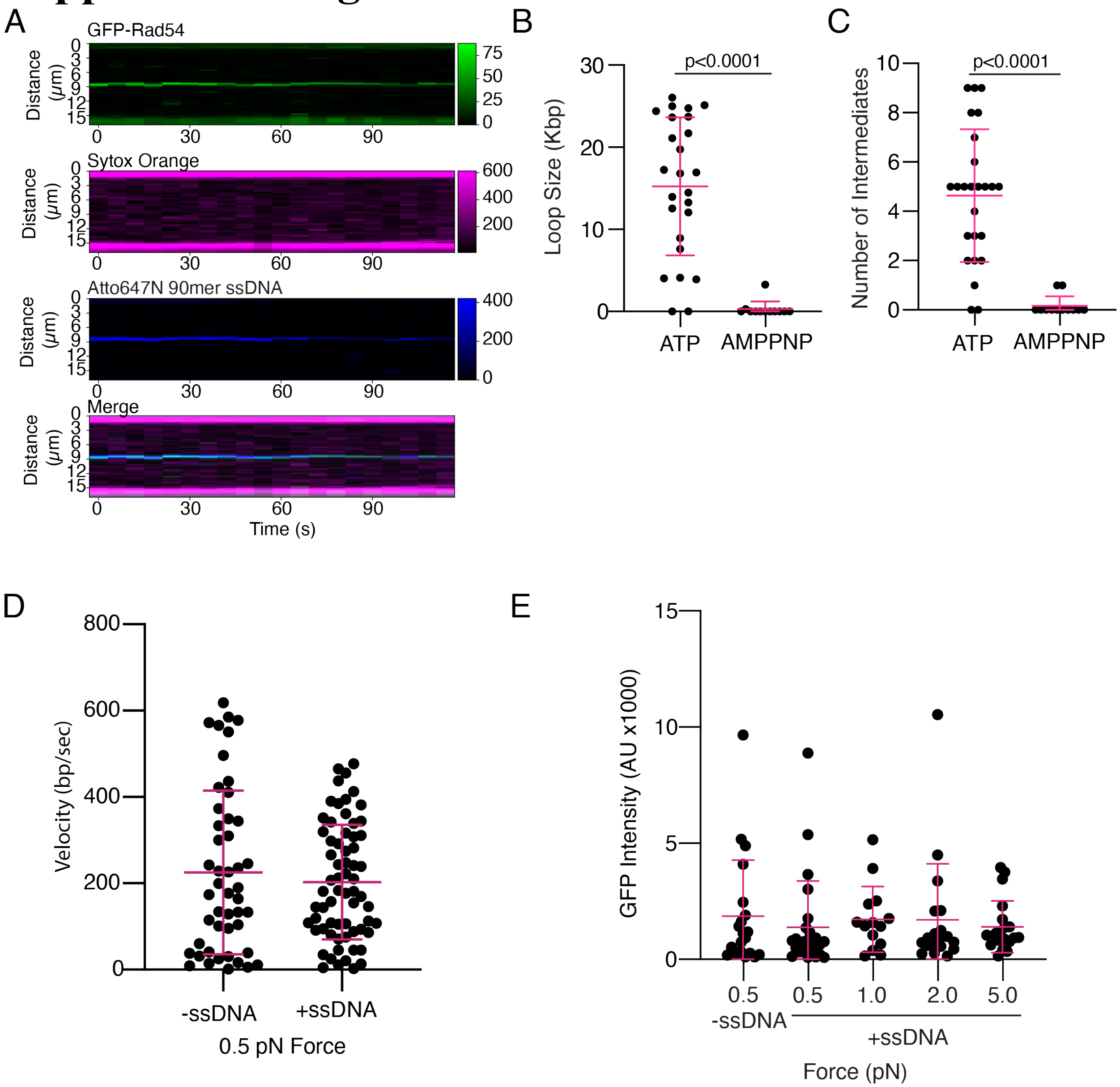

Supplemental Fig. S1: DNA compaction is limited without ATP hydrolysis and ssDNA
(A). Representative kymograph illustrating the binding of a PSC molecule in the presence of AMPPNP at 0.5 pN. Shown is the kymograph for GFP-Rad54 signal, Sytox Orange, Atto647 90-mer DNA, and a merged image. **(B).** A graph representing the initial loop size or amount of isolated DNA at 0.5 pN with ATP (N=25) and with AMPPNP (N=12). The bar represents the mean of the data, and the error bar represents the standard deviation of the data. The significance of the difference between the two samples was determined by a student's t-test. The data for the ATP case

are reproduced from Figure 1 **(C).** A graph quantifying the number of intermediates formed during compaction for ATP (N=25) and AMPPNP (N=12). The bar represents the mean of the data, and the error bars represent the standard deviation of the data. The significance of the data was determined by an unpaired t-test. The data for the ATP case were reproduced from Figure 1. **(D).** A graph comparing the velocities of DNA compaction in the absence (N=47) or presence (N=66) of 90-mer ssDNA. The crossbar represents the mean of the data, and the error bars represent the standard deviation. The significance of the difference between the two data sets was determined using a student's t-test. The +ssDNA data was reproduced from Figure 1. **(E)**. A graph comparing the GFP-Rad54 intensities for 0.5 (-ssDNA) (N=20), 0.5 (+ssDNA)(N=25), 1.0 (N=14), 2.0 (N=19), and 5.0 (N=20) pN of Force. The cross bar represents the mean of the data, and the error bars represent the standard deviation of the data.

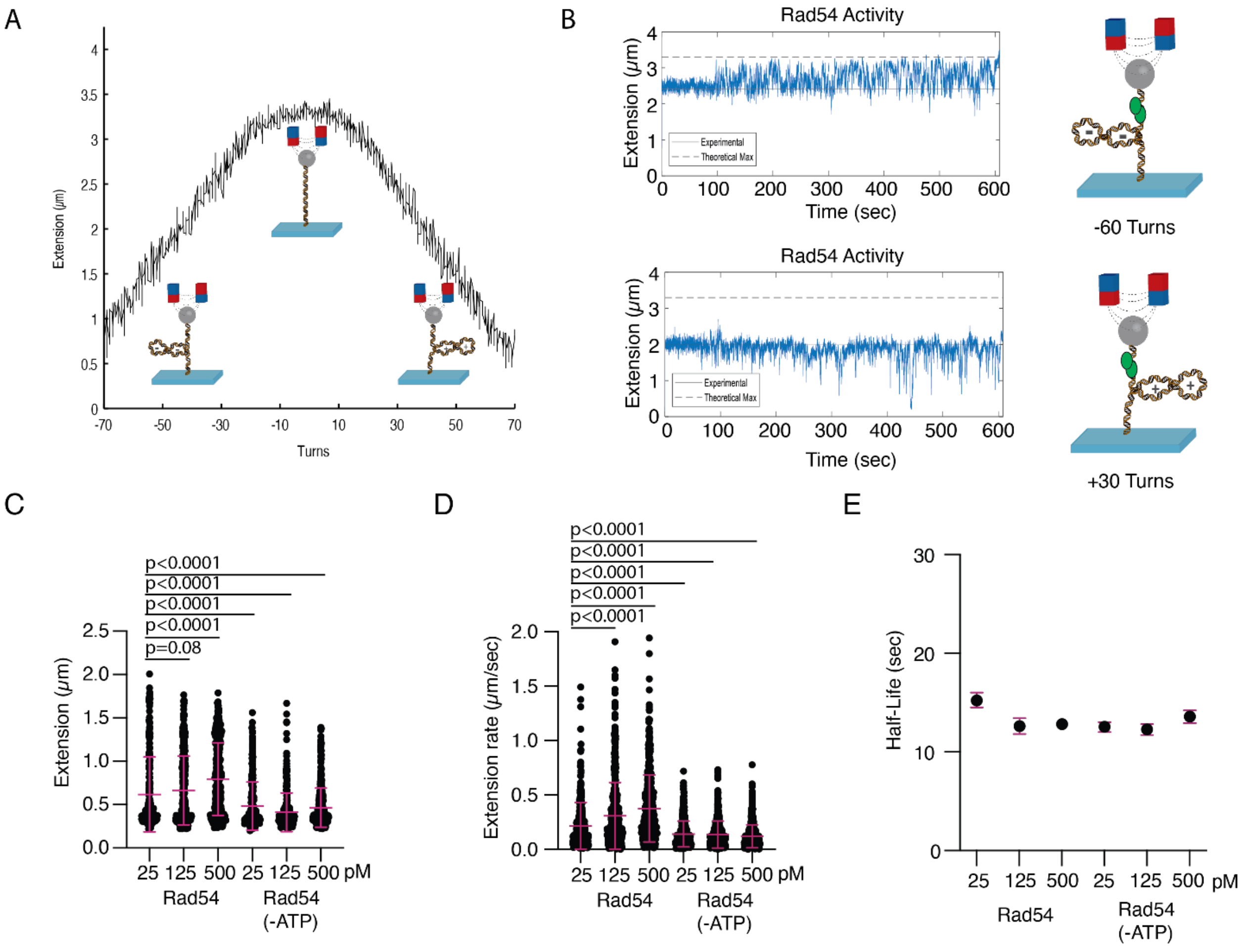

Supplemental Fig. S2: Rad54 enhances and stabilizes longer stretches of underwound DNA

(A). Example hat curve that illustrates the state of the DNA within each curve regime. At -60, the DNA forms a negative plectoneme, and at +30, a positive plectoneme. DNA at the apex is extended DNA. **(B).** Examples of Rad54 activity in the hat curve's -60 (Top) and +30 (Bottom) regions. The dashed line represents the max extension **(C).** Dot plot representing the extension of Rad54 +ATP at 25 (N=262), 125 (N=351), and 500 pM (N=350) and Rad54 -ATP at 25 (N=297), 125 (N=272), and 500 pM(N=466). The bar represents the mean, and the error bars represent the standard deviation. **(D).** Dot plot representing the extension per sec for Rad54 +ATP at 25 (N=388), 125

(N=462), and 500 pM (N=438) and Rad54 -ATP at 25 (N=464), 125 (N=409), 500 pM (N=693). The bar represents the mean, and the error bars represent the standard deviation of the data. **(E).** Graph representing the half-life of Rad54 at 25, 125, and 500 pM with and without ATP. The dots represent the half-life, and the bars represent the 95% confidence of the fit. The significance between groups in each data set was determined using an unpaired t-test.

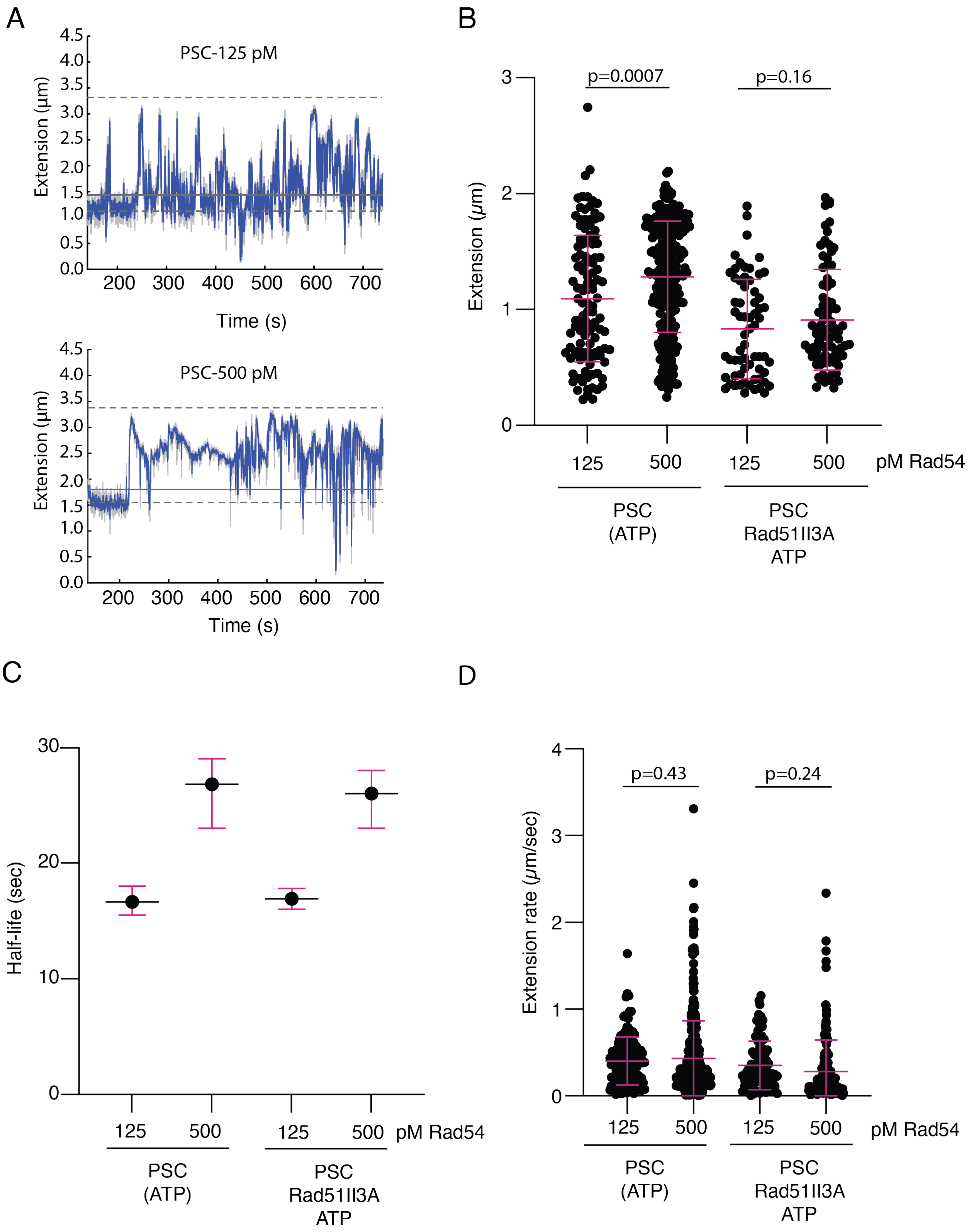
A
PSC-125 pM
Extension (μm)
Time (s)
PSC-500 pM
Extension (μm)
Time (s)
B
p=0.0007
p=0.16
Extension (μm)
125
500
125
500
pM Rad54
PSC
(ATP)
PSC
Rad51II3A
ATP
C
Half-life (sec)
125
500
125
500
pM Rad54
PSC
(ATP)
PSC
Rad51II3A
ATP
D
p=0.43
p=0.24
Extension rate (μm/sec)
125
500
125
500
pM Rad54
PSC
(ATP)
PSC
Rad51II3A
ATP

Supplemental Fig. S3: Impact of concentration on PSC activity

(A) Representative traces for the magnetic tweezer activity monitor for DNA molecules starting at -60 turns. The traces represent PSC conditions of 125 pM (Top) and 500 pM (Bottom). The top dashed line is the max extension, and the bottom dashed line is the DNA baseline. The solid line is the 3 standard deviations used as the cut-off for analysis. **(B).** Dot plot representing the extension added for PSCs at 125 pM (N=118) and 500 pM (N=265), and PSC with Rad51II3A at 125 pM (N=65) and 500 pM (N=82). The bar represents the mean of the data, and the error bars represent the standard deviation. **(C).** Graph representing the half-life measurements for PSC at 125 pM (N=120) and 500 pM (N=277), and PSC with Rad51II3A at 125 pM (N=65) and 500 pM (N=97). The dot represents the half-life, and the error bars represent the 95% confidence interval for the fit. **(D).** A graph representing the extension per second for PSC 125 pM (N=142) and 500 pM (N=427), and PSC with Rad51II3A at 125 pM (N=84) and 500 pM (N=157). The bar represents the mean of the data, and the error bars represent the standard deviation of the data. The significance of differences between all data was evaluated by an unpaired t-test. The 500 pM concentration was reproduced from Figure 4.

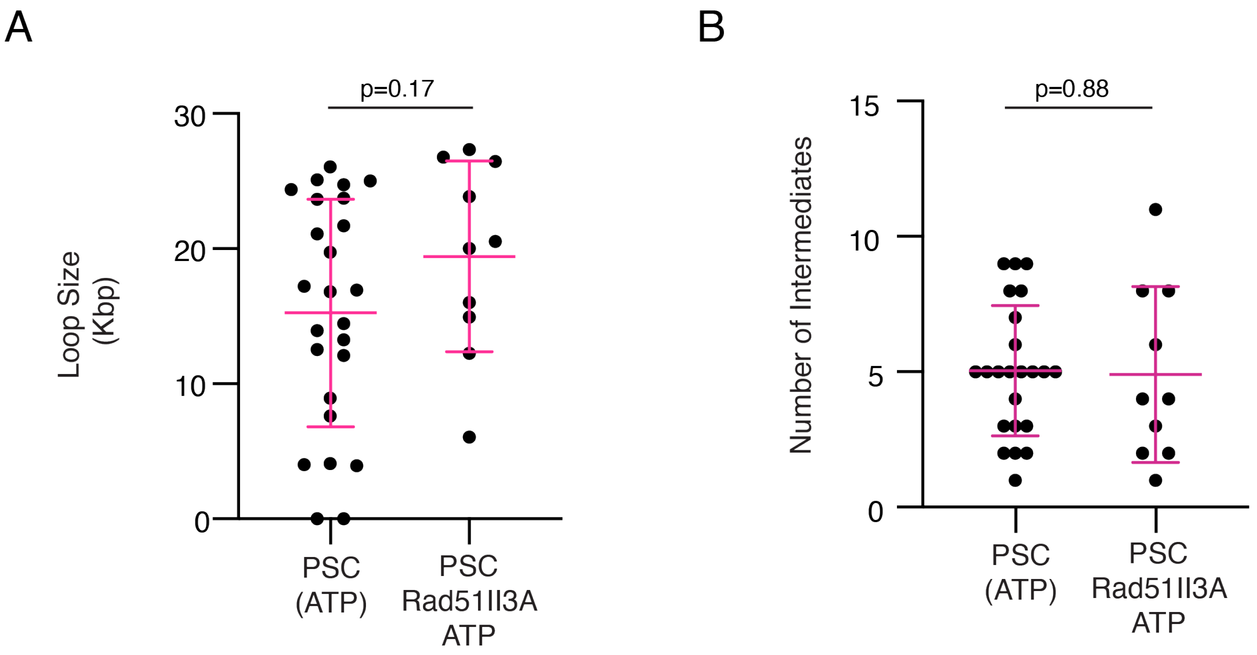

Supplemental Fig. S4: The donor DNA loop integrity is maintained in the Rad51II3A mutant (A). A graph representing the initial amount of DNA isolated in the PSC (N=25) and PSC with Rad51II3A (N=10). The bar represents the mean, and the error bars represent the standard deviation of the data. The significance of the difference between the two data sets was evaluated using a student's t-test. **(B).** A graph representing the number of intermediate contacts per tether PSC(N=23) and PSC with Rad51II3A (N=10). The bar represents the mean, and the error bars represent the standard deviation of the data. The significance of the difference between the two data sets was evaluated using a student's t-test.

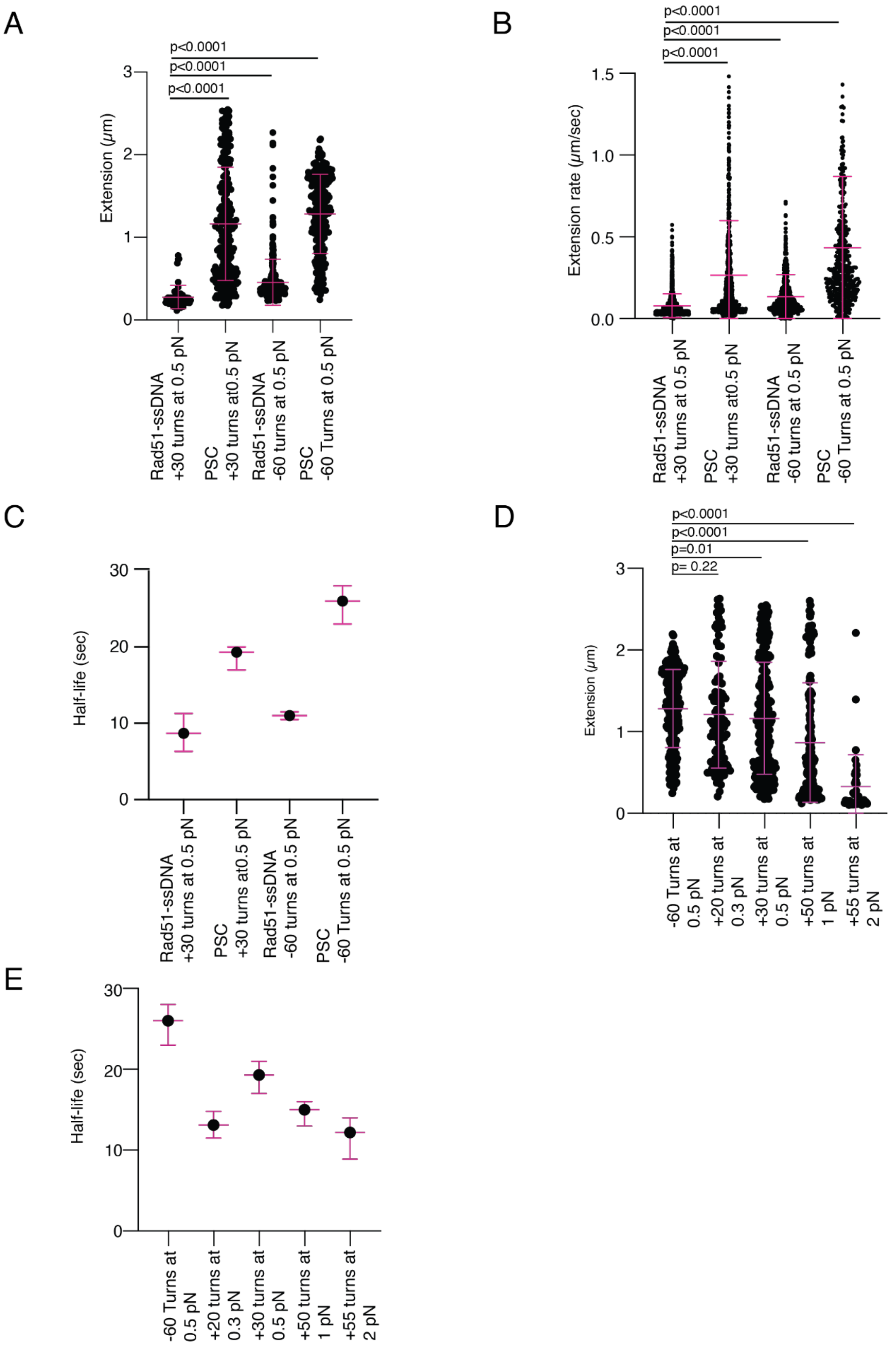
A
p<0.0001
p<0.0001
p<0.0001
Extension (μm)
Rad51-ssDNA +30 turns at 0.5 pN
PSC +30 turns at0.5 pN
Rad51-ssDNA -60 turns at 0.5 pN
PSC -60 Turns at 0.5 pN
B
p<0.0001
p<0.0001
p<0.0001
Extension rate (μm/sec)
Rad51-ssDNA +30 turns at 0.5 pN
PSC +30 turns at0.5 pN
Rad51-ssDNA -60 turns at 0.5 pN
PSC -60 Turns at 0.5 pN
C
Half-life (sec)
Rad51-ssDNA +30 turns at 0.5 pN
PSC +30 turns at0.5 pN
Rad51-ssDNA -60 turns at 0.5 pN
PSC -60 Turns at 0.5 pN
D
p<0.0001
p<0.0001
p=0.01
p= 0.22
Extension (μm)
-60 Turns at 0.5 pN
+20 turns at 0.3 pN
+30 turns at 0.5 pN
+50 turns at 1 pN
+55 turns at 2 pN
E
Half-life (sec)
-60 Turns at 0.5 pN
+20 turns at 0.3 pN
+30 turns at 0.5 pN
+50 turns at 1 pN
+55 turns at 2 pN

Supplemental Fig. S5: The PSC is more stable with negative turns added

(A). Extension measurements for Rad51-ssDNA alone at +30 turns and 0.5 pN (N=58), PSC at +30 turns and 0.5 pN (N=266), Rad51-ssDNA alone at -60 turns and 0.5 pN (N=309), and PSC at -60 turns and 0.5 pN (N=265). The bar represents the mean of the data, and the error bars represent the standard deviation of the data. Significance was determined by an unpaired t-test and the data for -60 turns and 0.5 pN is reproduced from other figures**. (B).** Extension rate measurements for Rad51-ssDNA alone at +30 turns and 0.5 pN (N=611), PSC at +30 turns and 0.5 pN (N=587), Rad51-ssDNA alone at -60 turns and 0.5 pN (N=580), and PSC at -60 turns and 0.5 pN (N=427). The bar represents the mean of the data, and the error bars represent the standard deviation of the data. Significance was determined by an unpaired t-test and the data for -60 turns and 0.5 pN is reproduced from other figures. **(C).** Half-life measurements for extension events Rad51-ssDNA alone at +30 turns and 0.5 pN, PSC at +30 turns and 0.5 pN, Rad51-ssDNA alone at -60 turns and 0.5 pN , and PSC at -60 turns and 0.5 pN. The dot represents the mean of the data, and the error bars represent the confidence in the fit. **(D).** Extension measurements for the PSC at -60 turns and 0.5 pN (N=265), +20 turns and 0.3 pN (N=104), +30 turns and 0.5 pN (N=266), +50 turns and 1.0 pN (N=176), and +55 turns at 2.0 pN (N=41). The bar represents the mean of the data, and the error bar represents the standard deviation. The significance was calculated from a student's t-test. The -60 and 0.5 pN data are reproduced from earlier figures **(E).** Half-life measurements for extension events for the PSC at -60 turns and 0.5 pN (N=265), +20 turns and 0.3 pN (N=104), +30 turns and 0.5 pN (N=265), +50 turns and 1.0 pN (N=176), and +55 turns at 2.0 pN (N=36). The dot represents the mean of the data, and the error bars represent the confidence in the fit.

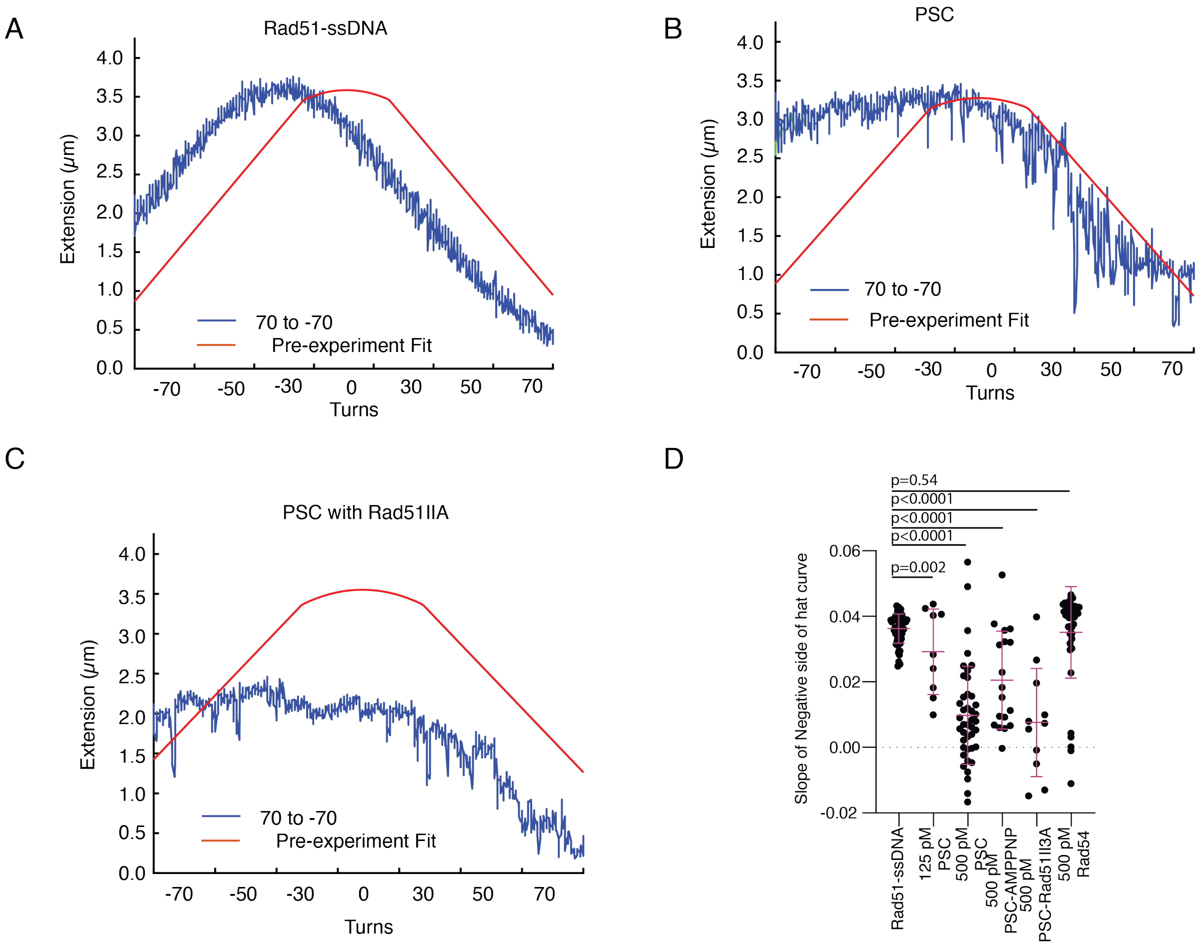

Supplemental Fig. S6: Post experiment hat curves show additional force added by Rad54

(A) Representative post-experiment hat curve for Rad51-ssDNA alone. The pre-hat curve is in Red, and the post experiment curve is in blue. **(B).** Representative post-experiment hat curve for the PSC. The pre-hat curve is in Red, and the post-experiment curve is in blue. **(C).** Representative post-experiment hat curve for PSC with Rad51II3A. The pre-hat curve is in Red, and the post-experiment curve is in blue. **(D).** Graph representing the slope of the -70 to -20 turns of the post-experiment hat curve for Rad51-ssDNA (N=59), PSC 125 pM (N=9), PSC 500 pM (N=42), PSC with AMPPNP (N=18), PSC with Rad51II3A 500 pM (N=11), and Rad54 alone 500 pM (N=44) The bar represents the mean, and the error bars the standard deviation of the data.

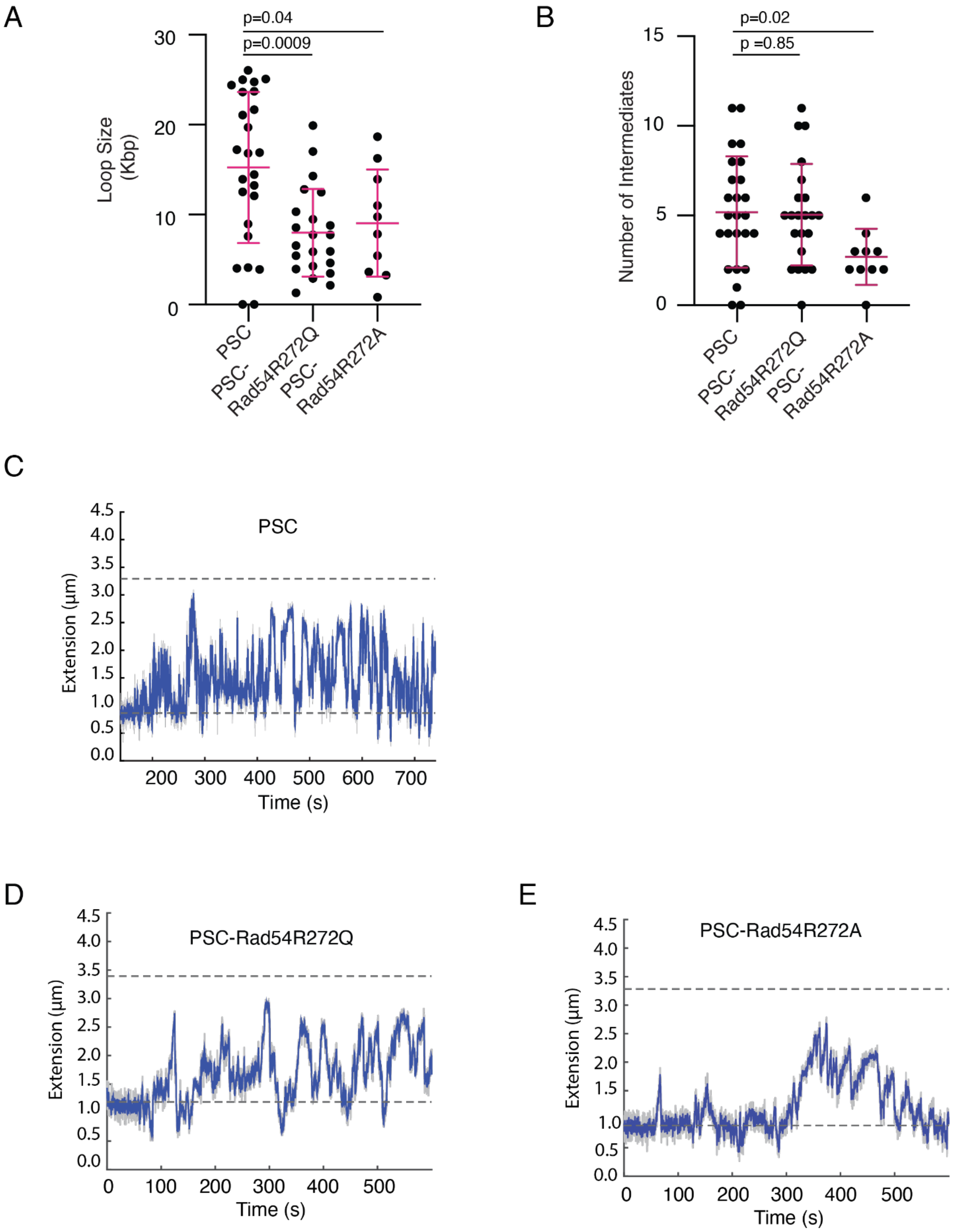
A
p=0.04
p=0.0009
Loop Size (Kbp)
30
20
10
0
PSC
PSC-Rad54R272Q
PSC-Rad54R272A
B
p=0.02
p =0.85
Number of Intermediates
15
10
5
0
PSC
PSC-Rad54R272Q
PSC-Rad54R272A
C
PSC
Extension (µm)
Time (s)
D
PSC-Rad54R272Q
Extension (µm)
Time (s)
E
PSC-Rad54R272A
Extension (µm)
Time (s)

Supplemental Fig. S7: Representative traces for MT experiments at 125 pM
(A). A graph representing the initial loop size for the PSC (N=25), PSC with Rad54R272Q (N=22), and PSC with Rad54R272A (N=10). The bar represents the mean, and the error bars the standard deviation of the data. The significance of differences was determined by an unpaired t-test. **(B).** Dot plot representing the number of isolated loops per tether for the PSC (N=25), PSC with Rad54R272Q (N=22), and PSC with Rad54R272A (N=10). The bar represents the mean, and the error bars represent the standard deviation of the data. The significance of differences was determined by a student's t-test. **(C).** Representative MT trace for PSC at 125 pM. **(D).** Representative MT trace for PSC with Rad54 R272Q at 125 pM**. (E).** Representative MT trace for PSC with Rad54 R272A at 125 pM. The top dashed line represents the maximum DNA extension, and the bottom dashed line represents the DNA baseline.
